# ER to synapse trafficking of NMDA receptors

**DOI:** 10.3389/fncel.2014.00394

**Published:** 2014-11-27

**Authors:** Martin Horak, Ronald S. Petralia, Martina Kaniakova, Nathalie Sans

**Affiliations:** ^1^Institute of Physiology, Academy of Sciences of the Czech Republic v.v.i.Prague, Czech Republic; ^2^Advanced Imaging Core, National Institute on Deafness and Other Communication Disorders, National Institutes of HealthBethesda, MD, USA; ^3^Neurocentre Magendie, Institut National de la Santé et de la Recherche Médicale, U862Bordeaux, France; ^4^Neurocentre Magendie, University of Bordeaux, U862Bordeaux, France

**Keywords:** glutamate receptor, excitatory neurotransmission, ion channel, internalization, intracellular trafficking, subcellular compartment

## Abstract

Glutamate is the major excitatory neurotransmitter in the mammalian central nervous system. There are three distinct subtypes of ionotropic glutamate receptors (GluRs) that have been identified including 2-amino-3-(5-methyl-3-oxo-1,2-oxazol-4-yl)propanoic acid receptors (AMPARs), *N*-methyl-D-aspartate receptors (NMDARs) and kainate receptors. The most common GluRs in mature synapses are AMPARs that mediate the fast excitatory neurotransmission and NMDARs that mediate the slow excitatory neurotransmission. There have been large numbers of recent reports studying how a single neuron regulates synaptic numbers and types of AMPARs and NMDARs. Our current research is centered primarily on NMDARs and, therefore, we will focus in this review on recent knowledge of molecular mechanisms occurring (1) early in the biosynthetic pathway of NMDARs, (2) in the transport of NMDARs after their release from the endoplasmic reticulum (ER); and (3) at the plasma membrane including excitatory synapses. Because a growing body of evidence also indicates that abnormalities in NMDAR functioning are associated with a number of human psychiatric and neurological diseases, this review together with other chapters in this issue may help to enhance research and to gain further knowledge of normal synaptic physiology as well as of the etiology of many human brain diseases.

## Introduction

The most common glutamate receptors (GluRs) in mature synapses are 2-amino-3-(5-methyl-3-oxo-1,2-oxazol-4-yl)propa­noic acid receptors (AMPARs) that mediate fast excitatory neurotransmission; *N*-methyl-D-aspartate receptors (NMDARs) serve mainly to modulate this neurotransmission by controlling the strength and number of AMPARs. However, during early postnatal development of many kinds of synapses, NMDARs predominate prior to the accumulation of AMPARs. The GluRs and their many associated proteins are “embedded” in an elaborate complex of interlinked proteins and cytoskeleton made by the postsynaptic density (PSD). The initial picture of the PSD was that of a static structure where GluRs were present, and were activated upon glutamate release. Over the last 10 years, a new picture of the PSD has emerged, that of a highly dynamic structure that increases or decreases in size and content during the entire life span of the individual, directly impacting spine shape. Such variations influence the physiological response of the postsynaptic side, the level of information storage and ultimately memory. For example, it is now believed that synapse function involves initial activation of AMPARs with glutamate binding, leading to a membrane depolarization that will release the magnesium block of the NMDAR channel, allowing calcium to enter the postsynaptic process via the glutamate-activated NMDAR. This calcium then initiates various metabolic pathways that ultimately can affect the strength of the synapse. Most commonly, these pathways involve various phosphorylation/dephosphorylation events that can activate or deactivate other pathways leading to changes in the strength or number of AMPARs at the synapse. Thus, it is clear that the trafficking and function of both types of GluRs, AMPARs and NMDARs, are tightly regulated by multiple unrelated mechanisms, ensuring that proper numbers and types of synaptic receptors are available in a given excitatory synapse. The interest in all these mechanisms has been strengthened by the recent discovery that pathologies such as Alzheimer’s disease and schizophrenia, but also mental retardation, fragile X syndrome, Rett syndrome, or Autism Syndrome Disorder, are due to disruption of synapse shape and function and not to structural permanent brain damage as initially thought. This has fostered new perspectives suggesting that by understanding how synapses form and are regulated, we could develop therapies to treat pathologies that were thought to be out of the reach of any curative intervention.

**Figure 1:**
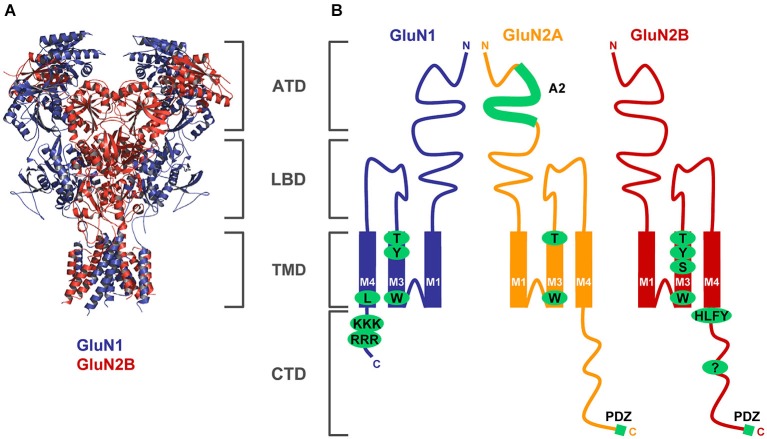
**Structural determinants regulating ER processing of functional NMDARs. (A)** The structure of the NMDAR heterotetramer, based on a recent paper by Gouaux’s laboratory, is indicated by blue (GluN1) and red (GluN2) colors (Lee et al., 2014). The membrane topology of the GluN subunits is described in detail in the Introduction section (ATD—amino-terminal domain, LBD—ligand-binding domain, TMD—transmembrane domain, CTD—C-terminal domain). **(B)** Schematic topology of the GluN subunits with indicated trafficking determinants (GluN1—several amino acid residues within the M3 domain and two ER retention signals, RRR and KKK, within the CTD; GluN2A—A2 segment within the ATD, several amino acid residues within the M3 domain; GluN2B—several amino acid residues within the M3 domain, HLFY export signal and unknown ER retention signal within the CTD). Both GluN2 subunits and some GluN1 splice variants (not shown) also contain PDZ-binding motifs in their far C-termini (see text for more details).

This review will focus on NMDARs. A functional NMDAR is a heterotetramer composed mainly of two GluN1 subunits and two GluN2 subunits; in some cases the GluN3 subunits are also incorporated into the heterotetramer (Petralia et al., 2009; Traynelis et al., 2010; Paoletti et al., 2013). Based on the crystallography structure of the recombinant NMDARs, recent studies showed that the functional GluN1/GluN2 heterotetramer is formed in a GluN1-2-1-2 subunit arrangement (i.e., 2 GluN1/GluN2 heterodimers combine to form the heterotetramer; Figure 1) (Karakas and Furukawa, 2014; Lee et al., 2014), although several other studies suggested that there is a GluN1-1-2-2 subunit arrangement in the NMDAR (Schorge and Colquhoun, 2003; Balasuriya et al., 2013). There are eight different GluN1 splice variants, four GluN2 subunits (GluN2A-D) and two GluN3 subunits (GluN3A-B) expressed in the mammalian CNS. All GluN subunits share similar membrane topology—four membrane domains (M1-M4), an extracellular N-terminus and a loop between M3 and M4 domains, and an intracellular C-terminus (Petralia et al., 2009). It is expected that the presence of relatively long N- and C-termini of the GluN subunits enables an NMDAR to dynamically interact with different proteins during its journey to the synapse, its retention at the synapse, and its removal from the synapse. The first step that shortly follows protein synthesis is the receptor subunit assembly that occurs in the endoplasmic reticulum (ER). Next, the receptors are processed in the Golgi apparatus and packaged in the Golgi complex by means of vesicles, which carry the GluRs to the membrane. They are subsequently internalized and reinserted at extrasynaptic sites before being anchored at the PSD. At each step of the trafficking process GluRs need to be associated with specific partners that allow the maturation and transport of the receptors. While significant progress has been made in identifying the proteins involved in anchoring GluRs at the PSD, little is known about the partners involved in the trafficking processes of these receptors. A deep knowledge of the trafficking from the ER to the membrane is paramount since these processes specify the final destination of the receptor complexes, and their deregulation can profoundly disrupt synaptic function. This review will highlight recent advancements in our understanding of early events in the trafficking of NMDARs as well as mechanisms regulating synaptic NMDARs. We will discuss the structural determinants and protein-protein interactions, both involved in the regulation of NMDARs, in three sections, summarizing; (1) events that happen early in the biosynthetic pathway (largely the ER); (2) events that happen in the transport of receptors after release from the ER; and (3) events that happen at the plasma membrane.

## Processing of NMDARs in the ER

What molecular mechanisms control the formation of the functional NMDAR heterotetramers in the ER? The GluN1 subunit is produced in the ER in large excess relative to GluN2 subunits, ensuring that sufficient amounts of GluN1 subunits are available for newly synthesized GluN2 and GluN3 subunits (Chazot and Stephenson, 1997; Huh and Wenthold, 1999). Several working models of the assembly of functional NMDARs in the ER have been proposed. First, several studies suggest that the GluN1-GluN1 and GluN2-GluN2 homodimers, which are initially formed, are required for the formation of the functional heterotetramers (Meddows et al., 2001; Schorge and Colquhoun, 2003; Papadakis et al., 2004; Qiu et al., 2005; Hansen et al., 2010). Second, another study proposed that the GluN1-GluN2 heterodimers are required for formation of heterotetrameric receptors (Schüler et al., 2008), or that GluN1-GluN1 homomers are the substrate for the oligomeric assembly of the heterotetramer (Atlason et al., 2007). The last model has been extended by a recent study that suggested that the N-terminal domains of the GluN1 subunits initially form homodimers and that the subsequent dimer dissociation is essential for the forming of the functional GluN1/GluN2 heteromers (Farina et al., 2011). Indeed, the reported promiscuity between the GluN1 and GluN2 N-terminal domains could explain the development of different working models of the assembly of NMDARs.

How does the cell ensure that only properly folded NMDAR heterotetramers are transported from the ER to the cell surface? In general, it is expected that the ER employs a quality control mechanism(s) for proteins exported from the ER to prevent the accumulation of unassembled and misfolded protein complexes via the trafficking pathways to the cell surface. In the case of NMDARs, it has been shown that all GluN2 subunits and some GluN1 splice variants are retained in the ER unless assembled (McIlhinney et al., 1998). The basic principle that the unassembled GluN subunits are retained in the ER has been demonstrated also in mice lacking the GluN1 subunit in the hippocampus, resulting in an accumulation of GluN2 subunits in the ER (Fukaya et al., 2003). Similarly, it has been reported that the GluN3A subunit requires the association with GluN1 subunits for its export from the ER (Perez-Otano et al., 2001).

What signals control ER retention of unassembled GluN subunits? Different regions of the GluN subunits have been proposed to regulate the assembly and/or ER retention of NMDA receptors (Figure 1). First, the C-termini of some GluN1 splice variants have been shown to contain two specific ER retention motifs, KKK and RRR, in the C1 cassette (Standley et al., 2000; Scott et al., 2001; Horak and Wenthold, 2009). Interestingly, the GluN1 variant, GluN1-3, which contains both the ER retention motifs in the C1 cassette and the PSD-95, Dlg, and Zo-1 (PDZ)-binding motif (-STVV) in the far C-terminus exhibits enhanced surface delivery even when expressed alone, suggesting that specific protein-protein interactions with other proteins such as the PSD-MAGUKs and COPII (which recognizes a divaline motif of the C-terminus of the GluN1-3) can regulate the ER retention of NMDAR subunits (Standley et al., 2000; Scott et al., 2001; Mu et al., 2003). The ER retention of the GluN1 subunit also can be modulated by phosphorylation by PKA and PKC of serine residues that are nearby the RRR motif, as shown using chimeric proteins of the C-terminus and single transmembrane proteins (tac = interleukin-2 receptor α subunit; CD8) (Scott et al., 2001); but our previous report did not confirm this observation using the full-length GluN1 constructs (Horak and Wenthold, 2009). The C-terminus of the GluN2B subunit attached to tac is retained in the ER, suggesting the presence of an ER retention signal (Hawkins et al., 2004). However, efforts to identify this specific signal have been unsuccessful, although truncation of the C-terminus appended to tac leads to increased surface expression in constructs containing the region up to residue 1070 of the GluN2B (Hawkins et al., 2004). This study also identified a short motif, HLFY, localized immediately after the M4 domain of the GluN2B subunits. This motif is likely required as an export signal from the ER for the properly folded NMDAR heterotetramers (Hawkins et al., 2004). However, a later study proposed that the HLFY motif is not necessary for the formation of the surface functional NMDAR, as it can be replaced by alanines if the C-terminus is absent (Yang et al., 2007). Similarly, the deletion of the GluN2B C-terminus including the HLFY motif did not affect the formation of functional receptors when two pieces of the GluN2B subunit, GluN2B truncated before M4 domain and GluN2B M4 domain, were co-expressed together with the GluN1 subunit (Horak et al., 2008a). Together, these data indicate that the HLFY motif may provide a structural role to ensure the proper orientation of the membrane domains and/or the C-termini in the ER processing of the GluN1/GluN2 receptors. Similarly, the GluN3B subunit may also use the RXR motifs for ER retention, which must be negated by the association with the GluN1 subunit (Matsuda et al., 2003).

The structures of the extracellular regions of the GluN subunits were also implicated in the regulation of ER processing of NMDARs. Specifically, Prof. Stephenson’s group reported that the structure of the glycine binding site in the GluN1 subunit is critical for the release of the functional NMDAR from the ER (Kenny et al., 2009). Similarly, another study revealed that the structure of the glutamate binding site within the GluN2B subunit controls the early processing of functional NMDARs (She et al., 2012). Given the fact that glutamate is likely present in the ER in the millimolar range (Berger et al., 1977; Meeker et al., 1989), it is plausible to speculate that a newly formed NMDAR heterotetramer is activated by agonists and then assessed for its proper functioning by a specific ER quality control machinery, as has been shown for the AMPARs (Penn et al., 2008). Future studies must resolve whether different affinities for glutamate and glycine among GluN1/GluN2A-D receptors, reported by many studies, regulate the ER processing of NMDARs (Traynelis et al., 2010). Furthermore, an ER retention signal has been identified in the A2 segment of the amino-terminal domain (ATD) of the GluN2A subunit; this must be masked by an interaction with the GluN1 subunit so that the functional NMDAR leaves the ER (Qiu et al., 2009). Interestingly, the appropriate region within the GluN2B subunit does not contain any ER retention signal, although there is relatively high sequence homology between the A2 segments of the GluN2A and GluN2B subunits. Because the identified A2 segment within the GluN2A subunit does not likely control the ER retention of unassembled GluN2A subunits, an additional ER retention signal(s) must exist in the remaining part of the GluN2A subunit (Qiu et al., 2009).

The structures of the membrane domains also likely regulate the ER processing of the functional NMDAR. Our previous reports identified critical structural determinants within the M3 domains of GluN1 and GluN2A-B subunits that cause the unassembled subunits to be retained in the ER (Horak et al., 2008b; Kaniakova et al., 2012a). However, we also showed that the structures of the M3 domains of the GluN1 and GluN2 subunits are critical for the release of the functional NMDARs from the ER, likely because the M3 domains mutually negate their ER retention signals (Horak et al., 2008b; Kaniakova et al., 2012a). Interestingly, these structural determinants within the M3 domains are present in the other glutamate receptor subtypes including their human variants as well, and thus it is likely that most ionotropic GluRs employ a common mechanism that includes specific inter-membrane domain interactions. This view is supported by recent studies showing that a specific amino acid residue within the GluN1 M4 domain regulates the early processing of NMDARs (Kaniakova et al., 2012b) and specific inter-membrane domain interactions of the M4 domain with the M1/M3 domains are required for surface expression of AMPARs (Salussolia et al., 2011). Moreover, recent data revealed that the M4 domain also controls the tetramerization of AMPARs (Salussolia et al., 2013). Whether the M4 domain also regulates tetramerization of NMDARs needs to be elucidated in future studies. Indeed, lack of precise structural information about the membrane regions of the NMDARs limits our current understanding of the processes that are involved in described phenomena.

Are the functional properties of NMDARs monitored during ER processing, as has been shown for AMPARs and kainate receptors (Priel et al., 2006; Penn et al., 2008)? As mentioned above, there are sufficient concentrations of glutamate present in the ER so that an NMDAR can be activated and monitored by the ER quality control machinery. The NMDARs are thought to have specific conformations associated with closed, open or desensitized states (Traynelis et al., 2010). Interestingly, the presence of the GluN2 subunit determines the functional and pharmacological properties of NMDARs, including their macroscopic desensitization, Mg^2+^ affinities and single-channel conductances (Traynelis et al., 2010; Paoletti, 2011; Siegler Retchless et al., 2012). But desensitization is not likely to be the major trafficking determinant of GluN1/GluN2B receptor subtype (Kaniakova et al., 2012a). Clearly, additional studies are necessary to elucidate molecular mechanisms that are behind the release of functional NMDARs from the ER. One of these mechanisms may include the ER resident chaperone protein, Sigma-1 receptor (σ-1R), which mediates trafficking of NMDARs to the cell surface (Pabba et al., 2014).

Most neurons express at least two of the most common GluN2 subunits, GluN2A and GluN2B, and thus three types of receptors can be formed, GluN1/GluN2A, GluN1/GluN2B and GluN1/GluN2A/GluN2B (Al-Hallaq et al., 2007; Tovar et al., 2013). The functional properties of these three receptor types are quite different (Hatton and Paoletti, 2005) and one may speculate that their formation is not due to the random association of the subunits, but is regulated by other factors such as developmental stage and synaptic activity. Interestingly, when GluN2A subunit increases its abundance at P7, the di-heteromeric GluN1/GluN2A and GluN1/GluN2B complexes are present in similar amounts to those seen in the later developmental stages (Al-Hallaq et al., 2007). This indicates that the formation of NMDAR complexes is not dependent only on the relative expression of the GluN2 subunits. A previous study also reported that there is a preference for association of the GluN2 subunits with different GluN1 splice variants (Sheng et al., 1994). Because the GluN1 variants containing the C2’ cassette exhibit an accelerated trafficking from the ER (Okabe et al., 1999; Mu et al., 2003; Horak and Wenthold, 2009) and neuronal activity leads to increased expression of C2’-containing GluN1 variants (Mu et al., 2003), it is obvious that the formation of individual NMDAR types and their exit from the ER are highly regulated processes that we are just learning to understand.

## From the exit of the ER to the synapse

### Trafficking of NMDA receptors from the ER to the plasma membrane

After being released from the ER, as for many other membrane proteins, NMDARs are further processed in the somatic Golgi apparatus and then distributed to the *trans* Golgi network (TGN) and endosomes, to finally reach the membrane and spines. While most NMDARs are likely processed in the cell body and then transported to the synapse, some use a non-conventional secretory pathway that bypasses the endoplasmic reticulum-Golgi intermediate compartment (ERGIC) pathway in the cell body and utilizes dendritic ER or Golgi outposts (Wenthold et al., 2003; Jeyifous et al., 2009). Indeed, neurons possess ramified dendrites that contain functional ER and Golgi outposts; even spines may contain such structures. NMDAR complexes seem to use different routes to reach the synapses, specifically bypassing or not the somatic Golgi apparatus.

### Maguks

**Figure 2:**
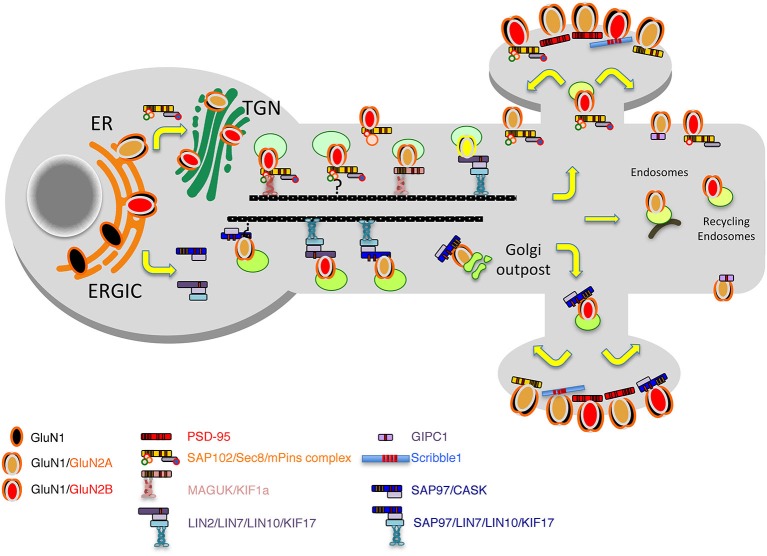
**Model of the secretory pathways used by NMDARs**. First, after their synthesis and export from the endoplasmic reticulum (ER), receptors travel through the Golgi before being inserted into vesicles and transported directly to the plasma membrane or transported into dendrites by vesicular transport by means of different complexes including the MAGUKs, exocyst and mPINS and kinesins. Alternatively, through an interaction with SAP97 and CASK, NMDAR can exit the ER, bypass the ERGIC pathway and travel in vesicles to dendritic Golgi outposts before reaching the membrane or the synapse.

**Figure 3:**
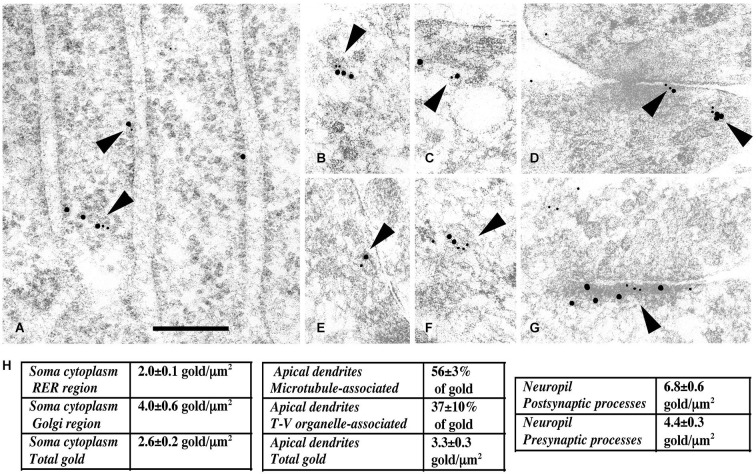
**Immunogold labeling of NMDARs, SAP102 and the exocyst complex. (A–G)** Double-immunogold labeling (arrowheads), in sections of the CA1 region of the hippocampus **(A)** stratum pyramidale; **(B–G)** stratum radiatum of juvenile animals, with antibodies to Sec6 or Sec8 (5 nm gold) and SAP102 or GluN2A/B (10 nm gold). **(A)** SAP102 and Sec8 in RER in a neuron soma. **(B)** SAP102 and Sec8 associated with a cytoplasmic tubulovesicular structure in a growth cone. **(C)** SAP102 and Sec8 associated with a small vesicle adjacent to a large one, in a large dendrite. **(D)** SAP102 and Sec8 at an early contact between neurites. **(E)** GluN2A/B and Sec8 associated with a cytoplasmic vesicle in a small neurite. **(F)** SAP102 and Sec6 associated with a cytoplasmic vesicle in a large dendrite. **(G)** SAP102 and Sec6 at a synapse on a dendrite shaft. Line scale is 200 nm. **(H)** Distribution of immunogold labeling (5 nm) for Sec8 in the CA1 stratum pyramidale/stratum radiatum region of the hippocampus at P10. Golgi region includes Golgi and the adjacent intermediate compartment and TGN. Microtubule-associated and tubulovesicular (T-V) organelle-associated categories are not mutually exclusive. Figure is a reprint of Figure 3 from Sans et al., 2003.

PDZ (PSD-95/Discs-large/ZO-1) domain-containing proteins, such as the MAGUK proteins, PSD-95, SAP102, and SAP97, were first identified as the major synaptic scaffolding proteins anchoring NMDARs at glutamatergic synapses (Lue et al., 1994; Kornau et al., 1995; Müller et al., 1995, 1996; Brenman et al., 1996; Kim et al., 1996; Lau et al., 1996; Niethammer et al., 1996; for review, see Sheng, 1996; Sheng and Kim, 1996; Kornau et al., 1997) but many studies have also implicated them in the trafficking of receptors to and/or from synapses (Wenthold et al., 2003; Elias and Nicoll, 2007). SAP102, a multiple PDZ domain with the same organization as PSD-95, got our attention very early because, according to early data, it is enriched both at synapses and in the general neuronal cytoplasm (Müller et al., 1996; Sans et al., 2000). Indeed, from the microsome fraction solubilized with Triton X-100, it was shown that GluN1 subunits could be immunoprecipated with SAP102 but not with PSD-95, showing that at least SAP102 could interact with NMDARs way before they reach the synapse (Standley et al., 2000). Therefore, we and others hypothesized that PDZ proteins in a more global way could be involved in the early events of assembly and delivery of receptors, and that these different events were regulated through interaction with other proteins (Figure 2). We performed yeast-two hybrid screens using the MAGUK, SAP102, as bait to identify novel regulators of GluR trafficking. A first screen with the three PDZ domains of SAP102 identified Sec8 as a potential partner of SAP102 (Sans et al., 2003). Sec8 is a member of the exocyst complex, with a previously uncharacterized PDZ-binding domain implicated in the secretory process (Hsu et al., 1996). The exocyst is a multiprotein complex containing eight proteins (Sec3, Sec5, Sec6, Sec8, Sec10, Sec15, Exo70, and Exo84) associated with intracellular compartments in yeast, and implicated in directing intracellular membrane vesicles through the secretory pathway to their sites of fusion with the plasma membrane (Hsu et al., 1999). However, its role in mammalian cells and the mechanisms by which this complex could move cargos are unclear. Using immunogold labeling in the CA1 *stratum pyramidale*/*stratum radiatum* region of the hippocampus at P10, we showed that Sec8 or Sec6 colocalized with SAP102 and NMDARs in the ER or Golgi region including the adjacent intermediate compartment and TGN (Figure 3). In the ER, a complex made up of NMDAR/SAP102 binds to Sec8 and some of the additional subunits of the complex such as Sec6 or Exo70. We showed that these interactions were important for surface delivery of the receptors in heterologous cells and for synaptic delivery in neurons (Sans et al., 2003). mPins was later shown to be involved in the proper folding of SAP102 complexes that participate in receptor trafficking (Sans et al., 2005). mPins interacts with G protein alpha-subunits (Gαi) and these interactions also play a role in the traffic of the receptors. The GDI activity of mPins can be overcome by the guanine nucleotide exchange factor, Resistance to inhibitors of cholinesterase (Ric-8A), which activates Gαi protein stimulating the release of Gαi-GTP (Tall and Gilman, 2005). It is possible that through this balanced action of mPins and Ric-8, Gαi proteins influence the traffic of NMDA receptors. Furthermore, intracellular NMDARs have been shown to colocalize with SAP102 by immunocytochemistry or immunogold labeling (Washbourne et al., 2004; Petralia et al., 2010; Standley et al., 2012). In addition, it has been shown that phosphorylation of Ser1480 on GluN2B can disrupt the interaction with SAP102 and PSD-95, thus leading to decreased targeting and anchoring of GluN2B in neurons (Chung et al., 2004). More recently, the hypothesis that SAP102 mediates trafficking of NMDARs has been strengthened by several studies. Indeed, neurons transfected with a ligand-binding deficient form of SAP102 show decreased synaptic clustering of NMDARs, although the SAP102 mutant forms were efficiently targeted to synapses (Minatohara et al., 2013). Interestingly, we showed in neurons transfected with full-length GluN2B and SAP102, using a switch in temperature from 37°C to 20°C to slow down the trafficking process, that SAP102 was colocalized with NMDARs at the level of the ER (Sans et al., 2005). Recently, Standley et al. confirmed these data, demonstrating that SAP102 interaction with NMDAR occurs very early in the secretory pathway; and using live imaging with Tac-GluN2A or Tac GluN2B chimeras, they showed that SAP102 first interacts with the tail of the receptors and that PSD-95 could also be involved in the traffic of some NMDARs in the post trans-Golgi network (Standley et al., 2012). It should be noted that the PSD-95 antibody used in this study (T60) also recognizes SAP97 (Sans et al., 2000) leaving open the possibility that both PSD-95 and SAP97 can interact with the chimeras. PSD-95 was also shown to interact with the exocyst (Riefler et al., 2003). It is interesting to note that the exocyst has also been involved in the trafficking of NMDAR–dependent AMPAR trafficking through a previously unidentified interaction between the Sec8 N-terminal sequence and GRIP1 (Mao et al., 2010). Exo70, another exocyst component, controls receptor synaptic accumulation (Gerges et al., 2006). All these results raised several interesting questions about the exact composition of the exocyst complex in the trafficking process, but they clearly show that the exocyst is implicated in delivery of receptors. SAP97 is another MAGUK with a high level of expression in the intracellular compartments (Sans et al., 2001), and that could interact with NMDARs (Niethammer et al., 1996; Songyang et al., 1997; Bassand et al., 1999). SAP97 is a GluA1 interactor involved in the precise targeting and clustering of AMPARs (Leonard et al., 1998; Sans et al., 2001; Nakagawa et al., 2004; Schlüter et al., 2006). In 2003, Di Luca et al. showed that SAP97 could indeed bind directly to GluN2A through its PDZ1 domain, and that this interaction is regulated by CaMKII (Gardoni et al., 2003; Mauceri et al., 2007). This interaction may be quite important in immature neurons since SAP97 seems to be able to drive the switch between GluN2B and GluN2A (Howard et al., 2010). It could also be involved in the trafficking of a subpopulation of receptors that do not use the conventional secretory pathway. Actually, Green et al. described a new path taken by some NMDARs associated with SAP97 and CASK and possibly KIF17 (Jeyifous et al., 2009). They showed that some NMDARs are directed from the somatic ER into a specialized dendrite ER subcompartment that targets NMDARs to dendritic Golgi outposts in dendrites. Later work showed that CASK regulates the conformation of SAP97, and thus is responsible for the specificity of SAP97 for AMPARs or NMDARs. In its compact conformation, SAP97 is preferentially associated with GluA1-containing AMPARs, while in the extended conformation due to CASK binding, SAP97 is associated with GluN2B-containing NMDARs (Lin et al., 2013). However, this is in contradiction to the initial finding showing that only GluN2A associates with non-phosphorylated SAP97 (Gardoni et al., 2003) and that GluN1 cannot interact with SAP97 (Leonard et al., 1998). These discrepancies could be due to SAP97 isoforms, which have distinctive roles in the trafficking of AMPARs and NMDARs. It was shown that the synaptic pool of AMPARs is regulated by αSAP97 while βSAP97 is important for the extrasynaptic pools of both AMPARs and NMDARs (Li et al., 2011).

### Kinesin molecular motors

The long distance transport along dendrites or axons depends on microtubules and motor proteins such as kinesins. KIF17 was the first kinesin involved in the trafficking of approximately 50 nm vesicles containing the GluN2B subunit of the NMDARs (Setou et al., 2000). KIF17 interacts with the PDZ domain of mLin10/Mint1/X11, which binds to GluN2B though an additional interaction with the adaptor proteins mLin2/CASK and mLin7/MALS/Velis. Additional work showed that these GluN2B containing vesicles move with a speed of 0.76 μm/sec (Guillaud et al., 2003). This is quite different from the 0.07 mm/sec found for GluN1 subunits (Washbourne et al., 2002) but could represent a different population of receptors. More recently, it has been shown that the interaction between KIF17 and its cargo is regulated by CaMKII (Guillaud et al., 2008) and that synaptic activity can control cargo itinerary (Hanus et al., 2014). Using loss of function experiments, Hirokawa et al. showed that the loss of GluN2B is compensated by an increase in GluN2A subunits at synapses suggesting that KIF17 is somehow specific for GluN2B subunits (Guillaud et al., 2003). However, in a *kif17* KO, both GluN2A and GluN2B subunits are reduced at synapses by 22% and 43% respectively, but by two different mechanisms. GluN2B transport is inhibited and GluN2A subunits are also lost due to an accelerated degradation by the ubiquitin-proteasome system (Yin et al., 2011). Interestingly, mLin2/CASK associates with SAP97 to regulate the traffic of Kir2 or NMDARs early in the secretory pathway (Leonoudakis et al., 2004; Jeyifous et al., 2009; Lin et al., 2013), suggesting that KIF17 may direct post-ER transport of GluN2B through different adaptor complexes including KIF17/Mint1/CASK/MALS and KIF17/Mint1/CASK/SAP97. Another possibility is that the KIF17/Mint1/CASK complex binds to SAP102 as well as SAP97. However, only 43% of the synaptic GluN2B subunits are lost in the *kif17* KO suggesting that other kinesins and complexes are also involved in GluN2B trafficking. Indeed, KIF1bα has been shown to interact directly with PSD-95 and SAP97 (Mok et al., 2002). Even though SAP102 was not tested in this paper, the C terminus sequence of KIF1bα (RETTV) contains a potential class I PDZ domain-binding motif, S/T-X-V (S/T, Ser or Thr; X, any amino acids; V, hydrophobic amino acids) that may interact with PSD-95 relatives such as PSD-93 and SAP102. In summary, SAP102, PSD-95 or SAP97 and Mint1/CASK have been implicated in the early trafficking of NMDARs (Figure 2). The data are so far insufficient to attach a specific MAGUK to a specific kinesin or subunit. It is clear that GluN2B has received more attention than GluN2A and may be more often associated with trafficking complexes.

### Myosins

KIF1b or KIF17 do not seem to enter directly into postsynaptic regions (Mok et al., 2002; Guillaud et al., 2003). Therefore, other means are needed to bring NMDAR to the PSD (Guillaud et al., 2008). It has been shown that the short distance transport inside the spine depends on actin and motor proteins such as myosins (Kapitein and Hoogenraad, 2011). Myosin Va or Vb and Myosin VI have been implicated in the trafficking of AMPARs (Wu et al., 2002; Osterweil et al., 2005; Lisé et al., 2006; Correia et al., 2008) but none of these have been involved in the regulation of NMDARs. While these are clearly involved in spine trafficking, other types of myosins (Lei et al., 2001; Amparan et al., 2005) may be involved in the delivery of NMDAR to the PSD. Other possible mechanisms for reaching the PSD include lateral diffusion along the extrasynaptic membrane (Choquet and Triller, 2013).

## Mechanisms regulating synaptic NMDARs

### NMDAR distribution and function at synapses and extrasynaptic regions of neurons

Literature on the function of NMDARs at synapses is extensive and we only can summarize it briefly in this review (see reviews: Petralia and Wenthold, 2008; Petralia et al., 2009; Traynelis et al., 2010; Paoletti et al., 2013; Sanz-Clemente et al., 2013b; Horak et al., 2014). The basic components of a synapse include: (1) the presynaptic terminal, which has a presynaptic membrane region called an active zone where synaptic vesicles dock to release glutamate into a synaptic cleft between the pre- and post-synaptic processes; and (2) the postsynaptic membrane, which contains the synaptic receptors that are bound to and/or associate with a complex of proteins that make up the postsynaptic density. Usually the postsynaptic process is either a dendrite shaft or a spine extending from a dendrite shaft. NMDARs are found on all parts of the synapse, including the pre- and postsynaptic membranes as well as extrasynaptic membrane areas that surround the synapse proper.

Typically, NMDARs of mature postsynaptic membranes are made of GluN1 combined with GluN2A or GluN2B, or both, as noted in the first section of this review. Other GluN2 subunits have more restricted distributions, such as GluN2C in the cerebellum and olfactory bulb and GluN2D in central parts of the forebrain and in the midbrain in mature animals. GluN2B and GluN2D are widespread in the embryonic and early postnatal brain. GluN3A is also a common NMDAR subunit in early postnatal development, while GluN3B appears mainly later in development. GluN2B and GluN2D may be the GluN2 subunits of the most common extrasynaptic NMDARs, and in fact, GluN2D may be exclusively extrasynaptic (Brickley et al., 2003; Harney et al., 2008). Mature neurons of the forebrain may show a prevalence for extrasynaptic NMDARs with GluN2B and synaptic ones with GluN2A (Tovar and Westbrook, 1999; reviewed in Hardingham and Bading, 2010; Gladding and Raymond, 2011; Parsons and Raymond, 2014), but other studies have not seen a clear delineation between preferential localization of these 2 subunits (Groc et al., 2006; Thomas et al., 2006; Harris and Pettit, 2007; Petralia et al., 2010). In addition, differences in GluN2A/GluN2B receptor composition in synapses have been found between left and right CA3 inputs onto CA1 pyramidal cells of the adult hippocampus (Shinohara et al., 2008). Synaptic spines that receive presynaptic terminals from the left CA3 (on both sides of the brain) are smaller and have a high density of GluN2B receptors, while spines receiving input from the right CA3 are larger and richer in GluN2A and GluA1 receptors.

Presynaptic NMDARs may be more widespread during neuronal differentiation and may be involved in guidance of the axonal growth cone (e.g., Wang et al., 2011; they also function at some developing and mature synapses (e.g., Jourdain et al., 2007; Larsen et al., 2011; Duguid, 2013)).

At the synapse, NMDARs are associated with scaffolding proteins of the postsynaptic density, especially the MAGUKs (PSD-95, PSD-93, SAP102, SAP97), as noted in the previous sections. The PDZ binding domain at the C-terminus of GluN2A and GluN2B binds to the first and the second PDZ domains of MAGUKs (Kornau et al., 1995; Niethammer et al., 1996). But NMDARs also may bind to MAGUKs via other domains (Cousins et al., 2009; Bard et al., 2010; Chen et al., 2011). PSD-95 and SAP102 are the main MAGUKs present in most mature forebrain synapses and both of them interact with GluN2A and GluN2B (Sans et al., 2000). PSD-95 is almost immobile in the PSD and forms an organized structure (Blanpied et al., 2008) maybe because of the presence of palmitoylation sites of the protein (El-Husseini et al., 2000); while SAP102 appears to be more widespread in the cytoplasm and extrasynaptic sites, in addition to its presence in the postsynaptic density (Müller et al., 1996; Sans et al., 2000, 2003; Standley et al., 2000). Furthermore, the majority of SAP102 in spines turns over within 5 min and its mobility is dependent on actin and glutamate receptor activation (Müller et al., 1996; Zheng et al., 2010, 2011). Indeed, during development, there may be a more prevalent association of SAP102 with GluN2B-containing NMDARs while PSD-95 may associate more with GluN2A (Sans et al., 2000; Petralia et al., 2005). In the superior colliculus and the visual cortex, after eye opening, synaptoneurosomal PSD-95 is bound to more GluN2A-rich NMDARs and less GluN2B-rich NMDARs, but the amount of the auxiliary protein, stargazin, bound to PSD-95, remains constant (Yoshii et al., 2003). In retinal ganglion cell synapses, GluN2A, the GluN1-c2’ variant, PSD-93, and PSD-95 are associated with the PSD, while GluN2B, the GluN1-c2 variant, and SAP102 tend to be perisynaptic (Zhang and Diamond, 2009). However, such a preferential association is not always clear, and mainly at mature synapses (Al-Hallaq et al., 2007; Petralia et al., 2010). In 2008, Nicoll et al. showed that SAP102 can traffic either GluN2A or GluN2B to the synapse, but PSD-95 selectively traffics GluN2A (Elias et al., 2008). Later, Groc et al. showed that this could be due to a specific divalent interaction (Bard et al., 2010). Other proteins may hold NMDARs in the synapse such as EphB receptors that associate with the extracellular N-terminus of NMDARs (Dalva et al., 2000); extracellular matrix proteins such as reelin may also affect NMDAR composition at synapses (Groc et al., 2007). NMDARs also can have an auxiliary subunit, Neto1 (complement C1r/C1s/Uegf/Bmp1 domain-containing neuropilin tolloid-like 1 protein), which may be important for synaptic plasticity (Ng et al., 2009). Electrophysiology and immunogold electron microscopy studies with Neto1 and Neto1/Neto2 knockout mice, respectively, found an increase in GluN2B-containing NMDARs at hippocampal CA3 mossy fiber synapses (Wyeth et al., 2014). Neto1 may form part of a trafficking complex that also includes NMDARs, MAGUKs and amyloid precursor protein (APP; Cousins et al., 2013).

**Figure 4:**
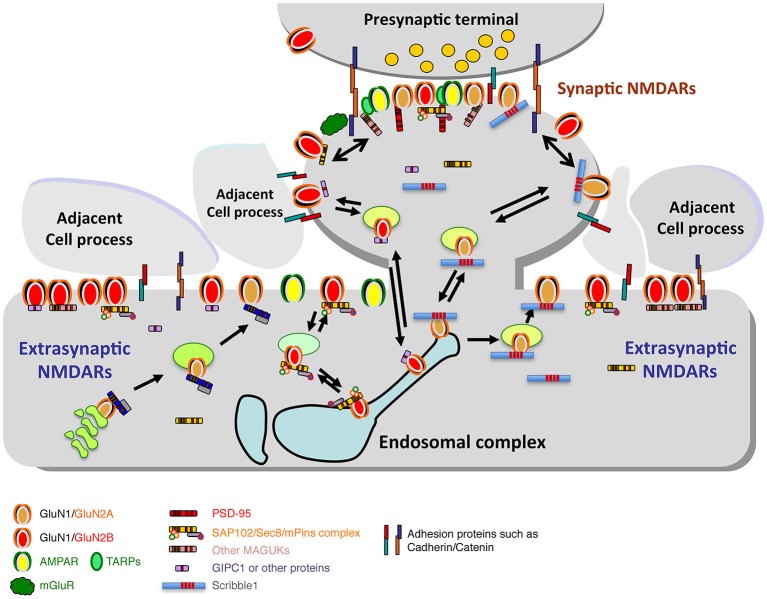
**Diagram illustrating the synaptic and extrasynaptic distributions of NMDARs and associated scaffolding and adhesion proteins, and especially the associations of extrasynaptic NMDARs with adjacent cell processes**. Note that other GluRs (AMPARs, kainate and delta iGluRs, and metabotropic GluRs (mGluRs)) are found at synapses and in extrasynaptic locations. AMPARs are typically the most abundant GluRs at synapses and may also be more common than NMDARs in extrasynaptic locations in some neurons. mGluRs are also widespread; some forms are particularly abundant in the perisynaptic zone. Like NMDARs, these GluRs also show close associations with other proteins that affect their trafficking and localization (not illustrated here). Trafficking of NMDARs through the Golgi pathway and/or endosomes is mediated by a number of associated proteins such as MAGUKs and Scribble1 (see text for details). Diagram and legend text is modified from Figure 4 of Petralia, 2012.

Extrasynaptic NMDARs may associate with scaffolding proteins also, including MAGUKs and GIPC (Figure 4; Yi et al., 2007; Petralia et al., 2010; reviewed in Gladding and Raymond, 2011; Petralia, 2012). However, most extrasynaptic NMDAR sites show little ultrastructural specialization, probably because there are relatively few proteins accumulated at these sites (Petralia et al., 2002, 2010; review: Petralia, 2012). Extrasynaptic NMDARs may be associated with a different set of proteins, involved in a different cell pathway, compared to NMDARs of the synapse. At least in some cases, activation of synaptic NMDAR pathways may upregulate neuronal cell functional plasticity and survival, while activation of extrasynaptic ones may turn on pathways leading to neurodegeneration (Liu et al., 2007; Hardingham and Bading, 2010; Gladding and Raymond, 2011; Bartlett and Wang, 2013; Karpova et al., 2013); and synaptic vs. extrasynaptic NMDARs also may be tied to pathways leading to LTP vs. long-term depression (LTD), respectively (Bartlett and Wang, 2013). In addition, NMDAR function is gated by different co-agonists, D-serine and glycine, in synaptic and extrasynaptic NMDARs, respectively (Papouin et al., 2012).

NMDAR function at synapses can be modified either by modulating the function of individual NMDAR complexes or by changing the composition or number of NMDARs in the synapse (see reviews: Rebola et al., 2010; Paoletti et al., 2013). The function of individual complexes can be modulated in many ways, including co-agonist activation, inhibition by extracellular zinc, as well as effects of polyamines and redox modulators. The C-terminus is subject to modulation via phosphorylation at several sites, and these can affect the strength of NMDAR-mediated currents and calcium permeability. Also, many G protein-coupled receptors, such as M1 muscarinic, LPA, metabotropic glutamate, and PACAP1 receptors enhance NMDAR function via phosphorylation events. Unfortunately, there is no room here to discuss these in any detail. Thus we will concentrate in the following sections only on changes in trafficking of NMDARs at synapses.

### Mechanisms of NMDAR movements: endocytosis and recycling

**Figure 5:**
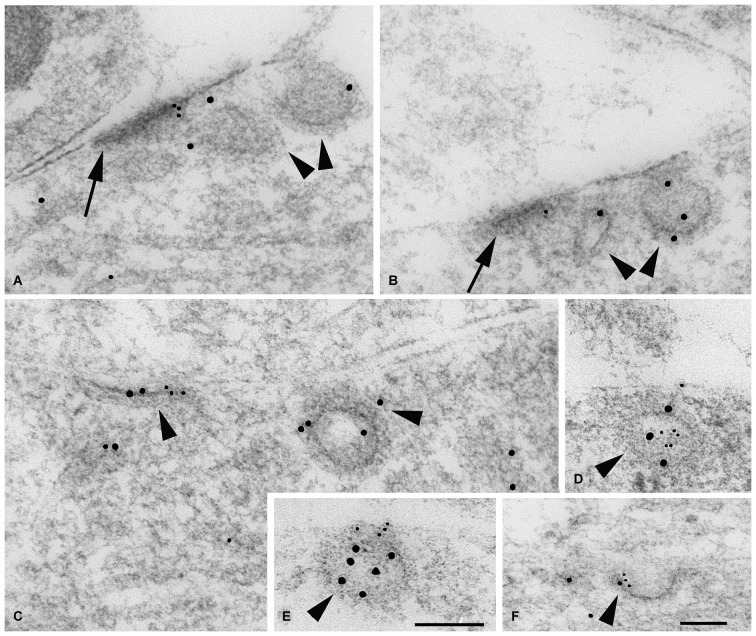
**Double immunogold labeling of clathrin-coated pits/vesicles (CCP/Vs; arrowheads) associated with bare densities (A,B; arrows) and extrasynaptic membrane regions (C–F) in the P2 hippocampus CA1 stratum radiatum with GluN1 (A–C,F) or GluN2A/B (D,E) antibody (5 nm gold), and clathrin (A–E) or adaptin α (F) antibody (10 nm gold). (A,B)** These two “bare” densities on dendrites actually show fairly close associations with adjacent processes. In both micrographs, a definitive CCP/V is seen in the vicinity of the density, and a second probable CCP/V is evident closer to the density. **(C,F)** In C, GluN1 and clathrin antibodies label an early, flat CCP/V adjacent to a CCP/V that is pinching off, and GluN1 and adaptin α label a better-developed CCP/V in F (both are dendrites). **(D,E)** GluN2A/B and clathrin antibodies label a newly formed CCV in **(E)**, and another CCV in **(D)** in a process at a point where the latter is contacted by another process. Scale bars, 100 nm. Scale in **(E)** is valid for micrographs **(A–E)**. Figure is a reprint of Figure 3 from Petralia et al., 2003.

While there may be distinct, relatively stable, functional populations of NMDARs in extrasynaptic locations as well as in the synapse, other extrasynaptic NMDARs may be more mobile—trafficking en route to or from a synapse (see previous section of this review, and reviews by Gladding and Raymond, 2011; Petralia, 2012; Paoletti et al., 2013). NMDARs destined for the synapse may exocytose at sites away from the synapse, either along the dendrite or on the sides of spines (Petralia et al., 2003; Washbourne et al., 2004), while endocytosis also may occur in these areas (Figure 5; Blanpied et al., 2002; Petralia et al., 2003; Rácz et al., 2004; Pérez-Otaño et al., 2006). Prior to synapse formation in early postnatal development, NMDARs appear to migrate to and from the cell surface in cycles of exo- and endocytosis (Washbourne et al., 2004). The dynamic movements of excitatory and inhibitory receptors involve constant switching between mobile and immobile states, depending on thermal agitation and the reversible binding to stable elements including scaffolding and cytoskeletal anchoring proteins, both in the postsynaptic density and in extrasynaptic sites (reviewed in Choquet and Triller, 2013). Most of the mobility studies of GluRs have involved AMPARs, but Groc et al. (2006), looking at NMDARs in dissociated hippocampal neuron cultures, found that the latter are more stable overall compared to AMPARs, with GluN2B-containing receptors having more surface mobility than those with GluN2A. Unlike AMPAR mobility, NMDAR mobility does not seem to be affected by TTX or KCl; thus, NMDARs may be more tightly attached to their surface positions (Groc et al., 2004). This is consistent with ideas of stable NMDAR populations both at synapses and in some extrasynaptic areas. Harris and Pettit (2007), using acute hippocampal slices, found little evidence of exchange of NMDARs between synaptic and extrasynaptic pools, which contained about 35% of the dendritic NMDARs. In contrast, Tovar and Westbrook (2002), using dissociated hippocampal neuron cultures, found an exchange of about 65% of synaptic NMDARs with extrasynaptic NMDARs in 7 min. Bard et al. (2010) also found evidence for rapid exchange of NMDARs in cultured hippocampal neurons.

Endocytosis of NMDARs utilizes clathrin-coated vesicles via association of the receptors with the AP-2 adaptor complex (Roche et al., 2001; Petralia et al., 2003; Lavezzari et al., 2004; Prybylowski et al., 2005); however, internalization may occur by an alternative, non-clathrin mediated endocytosis mechanism (Swanwick et al., 2009). Interaction of GluN2A with AP-2 is via a C-terminus dileucine motif (Lavezzari et al., 2004) although an additional AP-2 binding motif may be involved (Vissel et al., 2001). The AP-2 binding motif of GluN2B is YEKL, close to the C-terminus (Roche et al., 2001); MAGUK-dependent, fyn kinase-mediated phosphorylation of Tyr1472 in this motif prevents internalization and increases synaptic NMDAR currents (Prybylowski et al., 2005). NMDARs can take different pathways following their internalization and incorporation into early/sorting endosomes; thus, GluN2A-containing NMDARs preferentially traffic to late endosomes for degradation, while GluN2B-containing NMDARs tend to move to recycling endosomes from where they can return to the surface and to synapses (Lavezzari et al., 2004). Recently, we showed that Scribble1 could prevent GluN2A subunits from undergoing lysosomal trafficking and degradation by increasing their recycling to the plasma membrane following NMDAR activation (Piguel et al., 2014). We also showed that Arf6 and the Arf6-specific guanine nucleotide exchange factor (EFA6) are involved in that process. Interestingly, EFA6 is a partner of sorting nexin-1 (SNX1), a retromer component that is implicated in endosomal sorting and trafficking (Fukaya et al., 2014) and Scribble1 has also been shown to be implicated in the retromer localization to endosomes in epithelial cells (de Vreede et al., 2014). The retromer functions as a well-known complex involved in the retrograde transport from endosomes to the Golgi (Collins, 2008; Seaman et al., 2013) and is highly expressed in the hippocampus. Von Zastrow et al. recently showed that it is essential for functional surface expression of AMPARs and NMDARs at synapses (Choy et al., 2014). This function also may involve a complex of retromer and SNX27, which contains an N-terminal PDZ domain (Wang et al., 2013; Gallon et al., 2014). Moreover, many other proteins can affect NMDAR membrane expression differently and influence or change the ratio between GluN2A and GluN2B. These include PDZ proteins (i.e., MAGUKs) as already discussed (Losi et al., 2003; Sans et al., 2003, 2005; Chung et al., 2004; Mauceri et al., 2004; Howard et al., 2010), SNARE-related proteins (Lau et al., 2010; Suh et al., 2010) and kinases (Prybylowski et al., 2005; Sanz-Clemente et al., 2010, 2013a).

The mechanisms involved seem to be a bit different for GluN3 subunits. Endocytosis of GluN3A-containing NMDARs is mediated by PACSIN1 (protein kinase C and casein kinase substrate in neurons protein 1)/syndapin1, a neuron-specific accessory protein controlling clathrin-mediated endocytosis; PACSIN1 binds to the C-terminus of GluN3A via PACSIN1’s NPF motif (Pérez-Otaño et al., 2006). A novel endocytic motif (YWL) located within the cytoplasmic C-terminal tail of GluN3A is involved in the binding to the clathrin adaptor AP-2 (Chowdhury et al., 2013).

### Developmental changes in synaptic NMDAR number and composition

**Figure 6:**
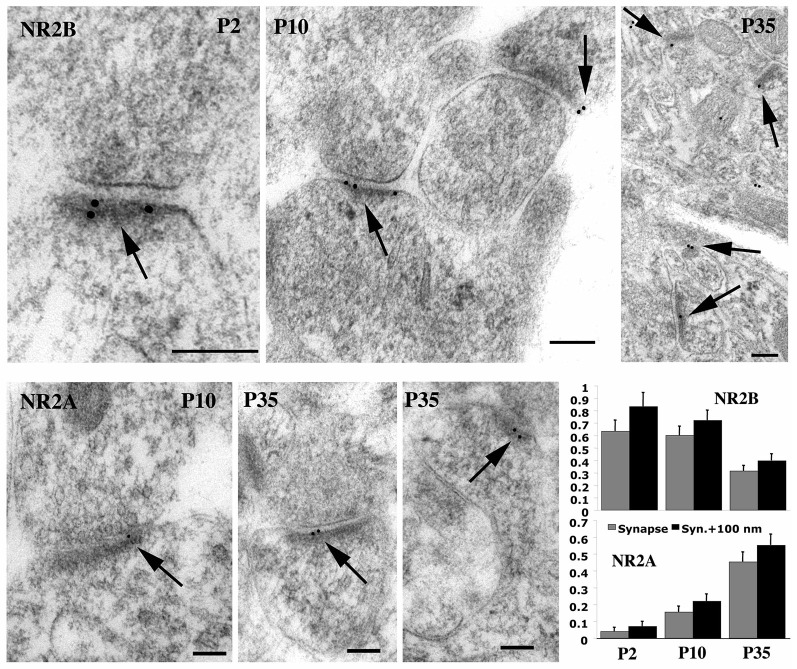
**Immunogold labeling for GluN2A (= NR2A) and GluN2B (= NR2B) in synapses during postnatal development of hippocampus CA1 stratum radiatum**. Micrographs illustrate the decrease in GluN2B and increase in GluN2A at synapses during development. For GluN2B, there was a significant decrease from P2 to P35 and from P10 to P35; 30%, 33%, and 23% of synapses were labeled for P2, P10, and P35, respectively. The Y-axis indicates gold per synapse (= synaptic cleft plus postsynaptic density) or per synapse + 100 nm (= 100 nm below the postsynaptic membrane). Scale bars for micrographs are 100 nm, arrows in micrographs indicate gold labeling associated with the postsynaptic density, and histograms show values plus standard errors. Figure is a reprint of Figure 6 from Petralia et al., 2005.

As we noted above, there are changes in NMDAR composition during development; NMDARs in early postnatal development mainly contain GluN2B or GluN2D, while GluN2A and GluN2C are more prevalent in adults. A downregulation of GluN2D-containing NMDARs in CA1 hippocampal pyramidal neurons may explain the great decrease in sensitivity to magnesium block beginning at P4 (Kirson et al., 1999). But especially there is a major switch from NMDARs with GluN2B to those with GluN2A. In the hippocampus, GluN2B is high at synapses at P2 (postnatal day 2) and there is a gradual decrease of GluN2B with age, as GluN2A increases; adults still show some NMDARs with GluN2B but GluN2A dominates (Figure 6); this also is accompanied by a similar switch in MAGUKs from mainly SAP102 to mainly PSD-95 plus some SAP102 (Sans et al., 2000; Petralia et al., 2005). This suggests that GluN2A-containing NMDARs are necessary for many functions found in the adult. In contrast, motoneurons in areas of the brain associated with the suckling reflex needed immediately after birth may already have high levels of GluN2A early in development (Oshima et al., 2002). The switch from GluN2B- to GluN2A-containing NMDARs is a conserved phenomenon among mammals and has been shown to occur in human development (Jantzie et al., 2013). In the rat hippocampus, activity induces a rapid change from GluN2B- to GluN2A-containing NMDARs, and this is bidirectional depending on activity (Bellone and Nicoll, 2007); in fact, studies of single synapses indicate that inactivity in silenced spine synapses enhances NMDAR currents and increases the number of GluN2B-containing NMDARs (Lee et al., 2010). At least in some cases, the switch is controlled by learning experiences such as with vision (light vs. dark rearing; Quinlan et al., 1999a,b). Stimuli that induce LTP induce the switch from GluN2B- to GluN2A-containing NMDARs in young animals (Bellone and Nicoll, 2007); this effect is not seen in older animals. As expected, regulation of the switch is controlled by calcium and phosphorylation. For example, casein kinase 2 (CK2) phosphorylates synaptic GluN2B to drive its endocytosis and replacement by GluN2A in cortical and hippocampal neuron cultures (Sanz-Clemente et al., 2010). The switch in hippocampal neurons actually involves many components, including activation of NMDARs and mGluR5, PLC, PKC, and calcium-release from IP3 receptor-dependent stores (Matta et al., 2011). Experience-dependent epigenetic remodeling associated with the GluN2B to GluN2A switch is mediated by a transcription factor called REST (repressor element 1 silencing transcription factor); when activated, REST is recruited to the promoter of the gene for GluN2B, where it binds to a 23-base pair motif in the promoter, recruiting co-repressors that can remodel the chromatin (Rodenas-Ruano et al., 2012).

Also as noted above, GluN3A is prevalent in early development and is lost or reduced with age. GluN3A is believed to prevent the stabilization of premature synapses, and its downregulation is necessary for synapse maturation (Wong et al., 2002; Pérez-Otaño et al., 2006; Roberts et al., 2009). GluN3A can be found in both the pre- and postsynaptic processes, and during development of the visual cortex, presynaptic NMDARs appear to switch from GluN1/2B/3A to GluN1/2B (Larsen et al., 2011). In this case, the GluN3-containing presynaptic NMDARs promote glutamate release and spike timing-dependent LTD in the juvenile visual cortex, probably important for developing the early receptive field properties. After maturation, the now GluN3A-lacking NMDARs may be active under strongly depolarizing conditions to promote the facilitation of repetitive stimuli. Possible differences in the development and distribution of GluN3A between rodents and humans have been reported (Eriksson et al., 2007; Nilsson et al., 2007). Indeed, human GluN3A contains a proline rich motif in the C-terminus that is not found in rat GluN3A; this domain may bind SH3 domains to affect trafficking, although apparently not the SH3 domain of PSD-95 (Eriksson et al., 2007). The developmental switch from GluN2B to GluN2C-containing NMDARs in cerebellar granule cells is mediated by innervation from mossy fibers that release neuregulin, activating ErbB2 and ErbB4 receptors on the granule cells (Ozaki et al., 1997; Garcia et al., 2000; Hahn et al., 2006). During this time, surface delivery of GluN2C-containing NMDARs depends on its phosphorylation by protein kinase B and its subsequent association with protein 14-3-3 (Chen and Roche, 2009).

### Changes in NMDAR number and composition in mature synapses

Generally, it has been thought that change in NMDAR composition at synapses is a developmental phenomenon accompanying the increase of AMPARs to adult levels (Sans et al., 2000; Petralia et al., 2005), and that adult plasticity involves changes in number and composition of AMPARs but not NMDARs (reviewed in Paoletti et al., 2013). Indeed, Bellone and Nicoll (2007) found no evidence of NMDAR plasticity in the CA1 region in hippocampal slices in 3 weeks postnatal Sprague-Dawley rats. A number of studies looking at NMDAR changes in the hippocampus of maturing rodents have concentrated on the period up to this point—from 2 to 3 weeks postnatal. Studies have shown evidence of bidirectional control of NMDAR GluN2A/GluN2B ratio in the Schaffer collateral/CA1 spine synapses of the hippocampus in slices from 2–3 week old Sprague-Dawley rats (Xu et al., 2009; Peng et al., 2010), i.e., looking at the period just prior to the 3 week limit indicated by Bellone and Nicoll (2007). Zhao et al. (2008) also provided evidence that stimulation can induce extrasynaptic NMDARs, mainly containing GluN2B (Figure 4), to move laterally into the synapse in 3 week-old hippocampal slices. In contrast to these studies showing changes in NMDARs only within 3 weeks postnatal, Grosshans et al. (Grosshans et al., 2002) found that LTP involves rapid PKC and Src-family dependent surface expression of NMDARs in the CA1 region of hippocampal slices from 6–8 week old rats. The reason for the difference is not clear but it may be that the latter study noted changes in extrasynaptic receptors. In support of this, studies of the visual cortex have indicated that there are later changes in extrasynaptic or presynaptic NMDARs (Yashiro et al., 2005; Larsen et al., 2011). Also, Harney et al. (2008) suggest that in the hippocampal dentate gyrus of 3–4 weeks old rats, extrasynaptic GluN2D-containing NMDARs are recruited to synapses during LTP. They presume that these receptors may be perisynaptic and that addition of these receptors to the synapse is a transient phenomenon; this is in contrast to other studies suggesting that GluN2D-containing NMDARs are exclusively extrasynaptic, as discussed above. Other studies in the 3–4 week period have shown the PKC-dependent insertion of NMDARs into mossy fiber synapses on CA3 hippocampal neurons during mossy fiber LTP (Kwon and Castillo, 2008) and orexin-induced, PLC/PKC-dependent insertion of NMDARs in synapses of dopaminergic neurons in the ventral tegmental area (Borgland et al., 2006). The latter synapses undergo the GluN2B to GluN2A switch in the first postnatal week, and interestingly, after this (and possibly even in adult mice), cocaine can evoke a switch to quasi-calcium impermeable NMDARs containing GluN3A and GluN2B (along with a switch to calcium-permeable AMPARs; Yuan et al., 2013). Subsequent recovery appears to be mediated by mGluR1, replacing the GluN3A-containing NMDARs with ones containing GluN2A again.

## Concluding remarks

In this brief review, we have highlighted some of the recent work on NMDAR assembly, ER exit to membrane and synapse trafficking of NMDARs. Because of its involvement in a critical function such as neurotransmission in a complex cell like a neuron, it is reasonable to propose that an NMDAR will interact with tens or maybe even hundreds of different proteins during its lifetime. Most of the identified interactions involve the cytoplasmic C-terminus of the GluN subunits. It is clear that more protein partners of NMDARs, including those that bind to extracellular and transmembrane domains (TMDs), await identification because they are not readily detectable using current assays, but may prove to be important to the trafficking and/or function of NMDARs. Furthermore, it is obvious that most studies dealing with the NMDARs have been performed using rat/mouse genes and thus the potential differences in trafficking of rodent and human NMDARs have been mostly neglected. However, human NMDARs exhibit similar functional and pharmacological properties to rodent NMDARs, consistent with the critical role that these receptor play in excitatory synapses (Hedegaard et al., 2012). Therefore, we expect that future studies will identify novel protein partners of the NMDARs as well as will address where the protein interactions of the NMDARs occur and how these interactions are regulated. Indeed, this knowledge will shed new light on our understanding of the different stages of processing, synaptic delivery, synaptic retention, and degradation of NMDARs and will also enable us to find new strategies to treat some human brain disorders.

## Conflict of interest statement

The authors declare that the research was conducted in the absence of any commercial or financial relationships that could be construed as a potential conflict of interest.

## References

[B1] Al-Hallaq R. A. Conrads T. P. Veenstra T. D. Wenthold R. J. (2007). NMDA di-heteromeric receptor populations and associated proteins in rat hippocampus. J. Neurosci. 27, 8334–8343. 10.1523/jneurosci.2155-07.2007 17670980 PMC2263005

[B2] Amparan D. Avram D. Thomas C. G. Lindahl M. G. Yang J. Bajaj G.. (2005). Direct interaction of myosin regulatory light chain with the NMDA receptor. J. Neurochem. 92, 349–361. 10.1111/j.1471-4159.2004.02869.x 15663482

[B3] Atlason P. T. Garside M. L. Meddows E. Whiting P. Mcilhinney R. A. (2007). N-Methyl-D-aspartate (NMDA) receptor subunit NR1 forms the substrate for oligomeric assembly of the NMDA receptor. J. Biol. Chem. 282, 25299–25307. 10.1074/jbc.m702778200 17606616

[B4] Balasuriya D. Goetze T. A. Barrera N. P. Stewart A. P. Suzuki Y. Edwardson J. M. (2013). α-Amino-3-hydroxy-5-methyl-4-isoxazole propionic acid (AMPA) and N-methyl-D-aspartate (NMDA) receptors adopt different subunit arrangements. J. Biol. Chem. 288, 21987–21998. 10.1074/jbc.M113.469205 23760273 PMC3724652

[B5] Bard L. Sainlos M. Bouchet D. Cousins S. Mikasova L. Breillat C.. (2010). Dynamic and specific interaction between synaptic NR2-NMDA receptor and PDZ proteins. Proc. Natl. Acad. Sci. U S A 107, 19561–19566. 10.1073/pnas.1002690107 20974938 PMC2984211

[B6] Bartlett T. E. Wang Y. T. (2013). The intersections of NMDAR-dependent synaptic plasticity and cell survival. Neuropharmacology 74, 59–68. 10.1016/j.neuropharm.2013.01.012 23357336

[B7] Bassand P. Bernard A. Rafiki A. Gayet D. Khrestchatisky M. (1999). Differential interaction of the tSXV motifs of the NR1 and NR2A NMDA receptor subunits with PSD-95 and SAP97. Eur. J. Neurosci. 11, 2031–2043. 10.1046/j.1460-9568.1999.00611.x 10336672

[B8] Bellone C. Nicoll R. A. (2007). Rapid bidirectional switching of synaptic NMDA receptors. Neuron 55, 779–785. 10.1016/j.neuron.2007.07.035 17785184

[B9] Berger S. J. Carter J. C. Lowry O. H. (1977). The distribution of glycine, GABA, glutamate and aspartate in rabbit spinal cord, cerebellum and hippocampus. J. Neurochem. 28, 149–158. 10.1111/j.1471-4159.1977.tb07720.x 833589

[B10] Blanpied T. A. Kerr J. M. Ehlers M. D. (2008). Structural plasticity with preserved topology in the postsynaptic protein network. Proc. Natl. Acad. Sci. U S A 105, 12587–12592. 10.1073/pnas.0711669105 18723686 PMC2519044

[B11] Blanpied T. A. Scott D. B. Ehlers M. D. (2002). Dynamics and regulation of clathrin coats at specialized endocytic zones of dendrites and spines. Neuron 36, 435–449. 10.1016/s0896-6273(02)00979-0 12408846

[B12] Borgland S. L. Taha S. A. Sarti F. Fields H. L. Bonci A. (2006). Orexin A in the VTA is critical for the induction of synaptic plasticity and behavioral sensitization to cocaine. Neuron 49, 589–601. 10.1016/j.neuron.2006.01.016 16476667

[B13] Brenman J. E. Chao D. S. Gee S. H. McGee A. W. Craven S. E. Santillano D. R.. (1996). Interaction of nitric oxide synthase with the postsynaptic density protein PSD-95 and alpha1-syntrophin mediated by PDZ domains. Cell 84, 757–767. 10.1016/s0092-8674(00)81053-3 8625413

[B14] Brickley S. G. Misra C. Mok M. H. Mishina M. Cull-Candy S. G. (2003). NR2B and NR2D subunits coassemble in cerebellar Golgi cells to form a distinct NMDA receptor subtype restricted to extrasynaptic sites. J. Neurosci. 23, 4958–4966. 12832518 10.1523/JNEUROSCI.23-12-04958.2003 PMC6741215

[B15] Chazot P. L. Stephenson F. A. (1997). Biochemical evidence for the existence of a pool of unassembled C2 exon-containing NR1 subunits of the mammalian forebrain NMDA receptor. J. Neurochem. 68, 507–516. 10.1046/j.1471-4159.1997.68020507.x 9003035

[B16] Chen B. S. Roche K. W. (2009). Growth factor-dependent trafficking of cerebellar NMDA receptors via protein kinase B/Akt phosphorylation of NR2C. Neuron 62, 471–478. 10.1016/j.neuron.2009.04.015 19477150 PMC2716006

[B17] Chen B. S. Thomas E. V. Sanz-Clemente A. Roche K. W. (2011). NMDA receptor-dependent regulation of dendritic spine morphology by SAP102 splice variants. J. Neurosci. 31, 89–96. 10.1523/JNEUROSCI.1034-10.2011 21209193 PMC3030119

[B18] Choquet D. Triller A. (2013). The dynamic synapse. Neuron 80, 691–703. 10.1016/j.neuron.2013.10.013 24183020

[B19] Chowdhury D. Marco S. Brooks I. M. Zandueta A. Rao Y. Haucke V.. (2013). Tyrosine phosphorylation regulates the endocytosis and surface expression of GluN3A-containing NMDA receptors. J. Neurosci. 33, 4151–4164. 10.1523/JNEUROSCI.2721-12.2013 23447623 PMC3682218

[B20] Choy R. W. Park M. Temkin P. Herring B. E. Marley A. Nicoll R. A.. (2014). Retromer mediates a discrete route of local membrane delivery to dendrites. Neuron 82, 55–62. 10.1016/j.neuron.2014.02.018 24698268 PMC4029335

[B21] Chung H. J. Huang Y. H. Lau L. F. Huganir R. L. (2004). Regulation of the NMDA receptor complex and trafficking by activity-dependent phosphorylation of the NR2B subunit PDZ ligand. J. Neurosci. 24, 10248–10259. 10.1523/jneurosci.0546-04.2004 15537897 PMC6730169

[B22] Collins B. M. (2008). The structure and function of the retromer protein complex. Traffic 9, 1811–1822. 10.1111/j.1600-0854.2008.00777.x 18541005

[B23] Correia S. S. Bassani S. Brown T. C. Lisé M. F. Backos D. S. El-Husseini A.. (2008). Motor protein-dependent transport of AMPA receptors into spines during long-term potentiation. Nat. Neurosci. 11, 457–466. 10.1038/nn2063 18311135

[B24] Cousins S. L. Innocent N. Stephenson F. A. (2013). Neto1 associates with the NMDA receptor/amyloid precursor protein complex. J. Neurochem. 126, 554–564. 10.1111/jnc.12280 23621516

[B25] Cousins S. L. Kenny A. V. Stephenson F. A. (2009). Delineation of additional PSD-95 binding domains within NMDA receptor NR2 subunits reveals differences between NR2A/PSD-95 and NR2B/PSD-95 association. Neuroscience 158, 89–95. 10.1016/j.neuroscience.2007.12.051 18308477

[B26] Dalva M. B. Takasu M. A. Lin M. Z. Shamah S. M. Hu L. Gale N. W.. (2000). EphB receptors interact with NMDA receptors and regulate excitatory synapse formation. Cell 103, 945–956. 10.1016/s0092-8674(00)00197-5 11136979

[B27] de Vreede G. Schoenfeld J. D. Windler S. L. Morrison H. Lu H. Bilder D. (2014). The scribble module regulates retromer-dependent endocytic trafficking during epithelial polarization. Development 141, 2796–2802. 10.1242/dev.105403 25005475 PMC4197622

[B28] Duguid I. C. (2013). Presynaptic NMDA receptors: are they dendritic receptors in disguise? Brain Res. Bull. 93, 4–9. 10.1016/j.brainresbull.2012.12.004 23279913

[B29] El-Husseini A. E. Craven S. E. Chetkovich D. M. Firestein B. L. Schnell E. Aoki C.. (2000). Dual palmitoylation of PSD-95 mediates its vesiculotubular sorting, postsynaptic targeting and ion channel clustering. J. Cell Biol. 148, 159–172. 10.1083/jcb.148.1.159 10629226 PMC2156213

[B30] Elias G. M. Elias L. A. Apostolides P. F. Kriegstein A. R. Nicoll R. A. (2008). Differential trafficking of AMPA and NMDA receptors by SAP102 and PSD-95 underlies synapse development. Proc. Natl. Acad. Sci. U S A 105, 20953–20958. 10.1073/pnas.0811025106 19104036 PMC2634944

[B31] Elias G. M. Nicoll R. A. (2007). Synaptic trafficking of glutamate receptors by MAGUK scaffolding proteins. Trends Cell Biol. 17, 343–352. 10.1016/j.tcb.2007.07.005 17644382

[B32] Eriksson M. Nilsson A. Samuelsson H. Samuelsson E.-B. Mo L. Åkesson E.. (2007). On the role of NR3A in human NMDA receptors. Physiol. Behav. 92, 54–59. 10.1016/j.physbeh.2007.05.026 17617428

[B33] Farina A. N. Blain K. Y. Maruo T. Kwiatkowski W. Choe S. Nakagawa T. (2011). Separation of domain contacts is required for heterotetrameric assembly of functional NMDA receptors. J. Neurosci. 31, 3565–3579. 10.1523/JNEUROSCI.6041-10.2011 21389213 PMC3063151

[B34] Fukaya M. Fukushima D. Hara Y. Sakagami H. (2014). EFA6A, a guanine nucleotide exchange factor for Arf6, interacts with sorting nexin-1 and regulates neurite outgrowth. J. Neurochem. 129, 21–36. 10.1111/jnc.12524 24261326

[B35] Fukaya M. Kato A. Lovett C. Tonegawa S. Watanabe M. (2003). Retention of NMDA receptor NR2 subunits in the lumen of endoplasmic reticulum in targeted NR1 knockout mice. Proc. Natl. Acad. Sci. U S A 100, 4855–4860. 10.1073/pnas.0830996100 12676993 PMC153645

[B36] Gallon M. Clairfeuille T. Steinberg F. Mas C. Ghai R. Sessions R. B.. (2014). A unique PDZ domain and arrestin-like fold interaction reveals mechanistic details of endocytic recycling by SNX27-retromer. Proc. Natl. Acad. Sci. U S A 111, E3604–E3613. 10.1073/pnas.1410552111 25136126 PMC4156734

[B37] Garcia R. A. Vasudevan K. Buonanno A. (2000). The neuregulin receptor ErbB-4 interacts with PDZ-containing proteins at neuronal synapses. Proc. Natl. Acad. Sci. U S A 97, 3596–3601. 10.1073/pnas.97.7.3596 10725395 PMC16285

[B38] Gardoni F. Mauceri D. Fiorentini C. Bellone C. Missale C. Cattabeni F.. (2003). CaMKII-dependent phosphorylation regulates SAP97/NR2A interaction. J. Biol. Chem. 278, 44745–44752. 10.1074/jbc.m303576200 12933808

[B39] Gerges N. Z. Backos D. S. Rupasinghe C. N. Spaller M. R. Esteban J. A. (2006). Dual role of the exocyst in AMPA receptor targeting and insertion into the postsynaptic membrane. EMBO J. 25, 1623–1634. 10.1038/sj.emboj.7601065 16601687 PMC1440842

[B40] Gladding C. M. Raymond L. A. (2011). Mechanisms underlying NMDA receptor synaptic/extrasynaptic distribution and function. Mol. Cell. Neurosci. 48, 308–320. 10.1016/j.mcn.2011.05.001 21600287

[B41] Groc L. Choquet D. Stephenson F. A. Verrier D. Manzoni O. J. Chavis P. (2007). NMDA receptor surface trafficking and synaptic subunit composition are developmentally regulated by the extracellular matrix protein Reelin. J. Neurosci. 27, 10165–10175. 10.1523/jneurosci.1772-07.2007 17881522 PMC6672660

[B42] Groc L. Heine M. Cognet L. Brickley K. Stephenson F. A. Lounis B.. (2004). Differential activity-dependent regulation of the lateral mobilities of AMPA and NMDA receptors. Nat. Neurosci. 7, 695–696. 10.1038/nn1270 15208630

[B43] Groc L. Heine M. Cousins S. L. Stephenson F. A. Lounis B. Cognet L.. (2006). NMDA receptor surface mobility depends on NR2A-2B subunits. Proc. Natl. Acad. Sci. U S A 103, 18769–18774. 10.1073/pnas.0605238103 17124177 PMC1693737

[B44] Grosshans D. R. Clayton D. A. Coultrap S. J. Browning M. D. (2002). LTP leads to rapid surface expression of NMDA but not AMPA receptors in adult rat CA1. Nat. Neurosci. 5, 27–33. 10.1038/nn779 11740502

[B45] Guillaud L. Setou M. Hirokawa N. (2003). KIF17 dynamics and regulation of NR2B trafficking in hippocampal neurons. J. Neurosci. 23, 131–140. 12514209 10.1523/JNEUROSCI.23-01-00131.2003 PMC6742138

[B46] Guillaud L. Wong R. Hirokawa N. (2008). Disruption of KIF17-Mint1 interaction by CaMKII-dependent phosphorylation: a molecular model of kinesin-cargo release. Nat. Cell Biol. 10, 19–29. 10.1038/ncb1665 18066053

[B47] Hahn C. G. Wang H. Y. Cho D. S. Talbot K. Gur R. E. Berrettini W. H.. (2006). Altered neuregulin 1-erbB4 signaling contributes to NMDA receptor hypofunction in schizophrenia. Nat. Med. 12, 824–828. 10.1038/nm1418 16767099

[B48] Hansen K. B. Furukawa H. Traynelis S. F. (2010). Control of assembly and function of glutamate receptors by the amino-terminal domain. Mol. Pharmacol. 78, 535–549. 10.1124/mol.110.067157 20660085 PMC2981397

[B49] Hanus C. Kochen L. Tom Dieck S. Racine V. Sibarita J. B. Schuman E. M.. (2014). Synaptic control of secretory trafficking in dendrites. Cell Rep. 7, 1771–1778. 10.1016/j.celrep.2014.05.028 24931613 PMC5321479

[B50] Hardingham G. E. Bading H. (2010). Synaptic versus extrasynaptic NMDA receptor signalling: implications for neurodegenerative disorders. Nat. Rev. Neurosci. 11, 682–696. 10.1038/nrn2911 20842175 PMC2948541

[B51] Harney S. C. Jane D. E. Anwyl R. (2008). Extrasynaptic NR2D-containing NMDARs are recruited to the synapse during LTP of NMDAR-EPSCs. J. Neurosci. 28, 11685–11694. 10.1523/JNEUROSCI.3035-08.2008 18987204 PMC3844786

[B52] Harris A. Z. Pettit D. L. (2007). Extrasynaptic and synaptic NMDA receptors form stable and uniform pools in rat hippocampal slices. J. Physiol. 584, 509–519. 10.1113/jphysiol.2007.137679 17717018 PMC2277145

[B53] Hatton C. J. Paoletti P. (2005). Modulation of triheteromeric NMDA receptors by N-terminal domain ligands. Neuron 46, 261–274. 10.1016/j.neuron.2005.03.005 15848804

[B54] Hawkins L. M. Prybylowski K. Chang K. Moussan C. Stephenson F. A. Wenthold R. J. (2004). Export from the endoplasmic reticulum of assembled N-methyl-d-aspartic acid receptors is controlled by a motif in the c terminus of the NR2 subunit. J. Biol. Chem. 279, 28903–28910. 10.1074/jbc.m402599200 15102836

[B55] Hedegaard M. Hansen K. B. Andersen K. T. Bräuner-Osborne H. Traynelis S. F. (2012). Molecular pharmacology of human NMDA receptors. Neurochem. Int. 61, 601–609. 10.1016/j.neuint.2011.11.016 22197913 PMC3335988

[B56] Horak M. Al-Hallaq R. A. Chang K. Wenthold R. J. (2008a). Role of the fourth membrane domain of the NR2B subunit in the assembly of the NMDA receptor. Channels (Austin) 2, 159–160. 10.4161/chan.2.3.6188 18836292 PMC2889170

[B57] Horak M. Chang K. Wenthold R. J. (2008b). Masking of the endoplasmic reticulum retention signals during assembly of the NMDA receptor. J. Neurosci. 28, 3500–3509. 10.1523/JNEUROSCI.5239-07.2008 18367616 PMC6670593

[B58] Horak M. Seabold G. K. Petralia R. S. (2014). “Trafficking of glutamate receptors and associated proteins in synaptic plasticity,” in The Synapse: Structure and Function, eds Pickel V. Segal M. (New York: Elsevier), 221–279.

[B59] Horak M. Wenthold R. J. (2009). Different roles of C-terminal cassettes in the trafficking of full-length NR1 subunits to the cell surface. J. Biol. Chem. 284, 9683–9691. 10.1074/jbc.M807050200 19188369 PMC2665089

[B60] Howard M. A. Elias G. M. Elias L. A. Swat W. Nicoll R. A. (2010). The role of SAP97 in synaptic glutamate receptor dynamics. Proc. Natl. Acad. Sci. U S A 107, 3805–3810. 10.1073/pnas.0914422107 20133708 PMC2840522

[B61] Hsu S. C. Hazuka C. D. Foletti D. L. Scheller R. H. (1999). Targeting vesicles to specific sites on the plasma membrane: the role of the sec6/8 complex. Trends Cell Biol. 9, 150–153. 10.1016/s0962-8924(99)01516-0 10203793

[B62] Hsu S. C. Ting A. E. Hazuka C. D. Davanger S. Kenny J. W. Kee Y.. (1996). The mammalian brain rsec6/8 complex. Neuron 17, 1209–1219. 10.1016/s0896-6273(00)80251-2 8982167

[B63] Huh K. H. Wenthold R. J. (1999). Turnover analysis of glutamate receptors identifies a rapidly degraded pool of the N-methyl-D-aspartate receptor subunit, NR1, in cultured cerebellar granule cells. J. Biol. Chem. 274, 151–157. 10.1074/jbc.274.1.151 9867823

[B64] Jantzie L. L. Talos D. M. Jackson M. C. Park H.-K. Graham D. A. Lechpammer M.. (2013). Developmental expression of *N*-methyl-D-aspartate (NMDA) receptor subunits in human white and gray matter: potential mechanism of increased vulnerability in the immature brain. Cereb. Cortex [Epub ahead of print]. 10.1093/cercor/bht246 24046081 PMC4303802

[B65] Jeyifous O. Waites C. L. Specht C. G. Fujisawa S. Schubert M. Lin E. I.. (2009). SAP97 and CASK mediate sorting of NMDA receptors through a previously unknown secretory pathway. Nat. Neurosci. 12, 1011–1019. 10.1038/nn.2362 19620977 PMC2779056

[B66] Jourdain P. Bergersen L. H. Bhaukaurally K. Bezzi P. Santello M. Domercq M.. (2007). Glutamate exocytosis from astrocytes controls synaptic strength. Nat. Neurosci. 10, 331–339. 10.1038/nn1849 17310248

[B67] Kaniakova M. Krausova B. Vyklicky V. Korinek M. Lichnerova K. Vyklicky L.. (2012a). Key amino acid residues within the third membrane domains of NR1 and NR2 subunits contribute to the regulation of the surface delivery of N-methyl-D-aspartate receptors. J. Biol. Chem. 287, 26423–26434. 10.1074/jbc.M112.339085 22711533 PMC3406725

[B68] Kaniakova M. Lichnerova K. Vyklicky L. Horak M. (2012b). Single amino acid residue in the M4 domain of GluN1 subunit regulates the surface delivery of NMDA receptors. J. Neurochem. 123, 385–395. 10.1111/jnc.12002 22937865

[B69] Kapitein L. C. Hoogenraad C. C. (2011). Which way to go? Cytoskeletal organization and polarized transport in neurons. Mol. Cell. Neurosci. 46, 9–20. 10.1016/j.mcn.2010.08.015 20817096

[B70] Karakas E. Furukawa H. (2014). Crystal structure of a heterotetrameric NMDA receptor ion channel. Science 344, 992–997. 10.1126/science.1251915 24876489 PMC4113085

[B71] Karpova A. Mikhaylova M. Bera S. Bär J. Reddy P. P. Behnisch T.. (2013). Encoding and transducing the synaptic or extrasynaptic origin of NMDA receptor signals to the nucleus. Cell 152, 1119–1133. 10.1016/j.cell.2013.02.002 23452857

[B72] Kenny A. V. Cousins S. L. Pinho L. Stephenson F. A. (2009). The integrity of the glycine co-agonist binding site of N-methyl-D-aspartate receptors is a functional quality control checkpoint for cell surface delivery. J. Biol. Chem. 284, 324–333. 10.1074/jbc.M804023200 18990687

[B73] Kim E. Cho K. O. Rothschild A. Sheng M. (1996). Heteromultimerization and NMDA receptor-clustering activity of Chapsyn-110, a member of the PSD-95 family of proteins. Neuron 17, 103–113. 10.1016/s0896-6273(00)80284-6 8755482

[B74] Kirson E. D. Schirra C. Konnerth A. Yaari Y. (1999). Early postnatal switch in magnesium sensitivity of NMDA receptors in rat CA1 pyramidal cells. J. Physiol. 521(Pt. 1), 99–111. 10.1111/j.1469-7793.1999.00099.x 10562337 PMC2269654

[B75] Kornau H. C. Schenker L. T. Kennedy M. B. Seeburg P. H. (1995). Domain interaction between NMDA receptor subunits and the postsynaptic density protein PSD-95. Science 269, 1737–1740. 10.1126/science.7569905 7569905

[B76] Kornau H. C. Seeburg P. H. Kennedy M. B. (1997). Interaction of ion channels and receptors with PDZ domain proteins. Curr. Opin. Neurobiol. 7, 368–373. 10.1016/s0959-4388(97)80064-5 9232802

[B77] Kwon H. B. Castillo P. E. (2008). Long-term potentiation selectively expressed by NMDA receptors at hippocampal mossy fiber synapses. Neuron 57, 108–120. 10.1016/j.neuron.2007.11.024 18184568 PMC2390917

[B78] Larsen R. S. Corlew R. J. Henson M. A. Roberts A. C. Mishina M. Watanabe M.. (2011). NR3A-containing NMDARs promote neurotransmitter release and spike timing-dependent plasticity. Nat. Neurosci. 14, 338–344. 10.1038/nn.2750 21297630 PMC3474337

[B79] Lau L. F. Mammen A. Ehlers M. D. Kindler S. Chung W. J. Garner C. C.. (1996). Interaction of the N-methyl-D-aspartate receptor complex with a novel synapse-associated protein, SAP102. J. Biol. Chem. 271, 21622–21628. 10.1074/jbc.271.35.21622 8702950

[B80] Lau C. G. Takayasu Y. Rodenas-Ruano A. Paternain A. V. Lerma J. Bennett M. V.. (2010). SNAP-25 is a target of protein kinase C phosphorylation critical to NMDA receptor trafficking. J. Neurosci. 30, 242–254. 10.1523/JNEUROSCI.4933-08.2010 20053906 PMC3397691

[B81] Lavezzari G. McCallum J. Dewey C. M. Roche K. W. (2004). Subunit-specific regulation of NMDA receptor endocytosis. J. Neurosci. 24, 6383–6391. 10.1523/jneurosci.1890-04.2004 15254094 PMC6729547

[B82] Lee C. H. Lü W. Michel J. C. Goehring A. Du J. Song X.. (2014). NMDA receptor structures reveal subunit arrangement and pore architecture. Nature 511, 191–197. 10.1038/nature13548 25008524 PMC4263351

[B83] Lee M. C. Yasuda R. Ehlers M. D. (2010). Metaplasticity at single glutamatergic synapses. Neuron 66, 859–870. 10.1016/j.neuron.2010.05.015 20620872 PMC2911980

[B84] Lei S. Czerwinska E. Czerwinski W. Walsh M. P. MacDonald J. F. (2001). Regulation of NMDA receptor activity by F-actin and mysoin light chain kinase. J. Neurosci. 21, 8464–8472. 11606635 10.1523/JNEUROSCI.21-21-08464.2001 PMC6762792

[B85] Leonard A. S. Davare M. A. Horne M. C. Garner C. C. Hell J. W. (1998). SAP97 is associated with the alpha-amino-3-hydroxy-5-methylisoxazole-4-propionic acid receptor GluR1 subunit. J. Biol. Chem. 273, 19518–19524. 10.1074/jbc.273.31.19518 9677374

[B86] Leonoudakis D. Conti L. R. Radeke C. M. McGuire L. M. Vandenberg C. A. (2004). A multiprotein trafficking complex composed of SAP97, CASK, Veli and Mint1 is associated with inward rectifier Kir2 potassium channels. J. Biol. Chem. 279, 19051–19063. 10.1074/jbc.m400284200 14960569

[B87] Li D. Specht C. G. Waites C. L. Butler-Munro C. Leal-Ortiz S. Foote J. W.. (2011). SAP97 directs NMDA receptor spine targeting and synaptic plasticity. J. Physiol. 589, 4491–4510. 10.1113/jphysiol.2011.215566 21768261 PMC3208220

[B88] Lin E. I. Jeyifous O. Green W. N. (2013). CASK regulates SAP97 conformation and its interactions with AMPA and NMDA receptors. J. Neurosci. 33, 12067–12076. 10.1523/JNEUROSCI.0816-13.2013 23864692 PMC3713737

[B89] Lisé M. F. Wong T. P. Trinh A. Hines R. M. Liu L. Kang R.. (2006). Involvement of myosin Vb in glutamate receptor trafficking. J. Biol. Chem. 281, 3669–3678. 10.1074/jbc.m511725200 16338934

[B90] Liu Y. Wong T. P. Aarts M. Rooyakkers A. Liu L. Lai T. W.. (2007). NMDA receptor subunits have differential roles in mediating excitotoxic neuronal death both in vitro and in vivo. J. Neurosci. 27, 2846–2857. 10.1523/jneurosci.0116-07.2007 17360906 PMC6672582

[B91] Losi G. Prybylowski K. Fu Z. Luo J. Wenthold R. J. Vicini S. (2003). PSD-95 regulates NMDA receptors in developing cerebellar granule neurons of the rat. J. Physiol. 548, 21–29. 10.1113/jphysiol.2002.034918 12576494 PMC2342804

[B92] Lue R. A. Marfatia S. M. Branton D. Chishti A. H. (1994). Cloning and characterization of hdlg: the human homologue of the Drosophila discs large tumor suppressor binds to protein 4.1. Proc. Natl. Acad. Sci. U S A 91, 9818–9822. 10.1073/pnas.91.21.9818 7937897 PMC44908

[B93] Mao L. Takamiya K. Thomas G. Lin D. T. Huganir R. L. (2010). GRIP1 and 2 regulate activity-dependent AMPA receptor recycling via exocyst complex interactions. Proc. Natl. Acad. Sci. U S A 107, 19038–19043. 10.1073/pnas.1013494107 20956289 PMC2973854

[B94] Matsuda K. Fletcher M. Kamiya Y. Yuzaki M. (2003). Specific assembly with the NMDA receptor 3B subunit controls surface expression and calcium permeability of NMDA receptors. J. Neurosci. 23, 10064–10073. 14602821 10.1523/JNEUROSCI.23-31-10064.2003 PMC6740865

[B95] Matta J. A. Ashby M. C. Sanz-Clemente A. Roche K. W. Isaac J. T. (2011). mGluR5 and NMDA receptors drive the experience- and activity-dependent NMDA receptor NR2B to NR2A subunit switch. Neuron 70, 339–351. 10.1016/j.neuron.2011.02.045 21521618 PMC3087383

[B96] Mauceri D. Cattabeni F. Di Luca M. Gardoni F. (2004). Calcium/calmodulin-dependent protein kinase II phosphorylation drives synapse-associated protein 97 into spines. J. Biol. Chem. 279, 23813–23821. 10.1074/jbc.m402796200 15044483

[B97] Mauceri D. Gardoni F. Marcello E. Di Luca M. (2007). Dual role of CaMKII-dependent SAP97 phosphorylation in mediating trafficking and insertion of NMDA receptor subunit NR2A. J. Neurochem. 100, 1032–1046. 10.1111/j.1471-4159.2006.04267.x 17156128

[B98] McIlhinney R. A. Le Bourdellès B. Molnár E. Tricaud N. Streit P. Whiting P. J. (1998). Assembly intracellular targeting and cell surface expression of the human N-methyl-D-aspartate receptor subunits NR1a and NR2A in transfected cells. Neuropharmacology 37, 1355–1367. 10.1016/s0028-3908(98)00121-x 9849671

[B99] Meddows E. Le Bourdelles B. Grimwood S. Wafford K. Sandhu S. Whiting P.. (2001). Identification of molecular determinants that are important in the assembly of N-methyl-D-aspartate receptors. J. Biol. Chem. 276, 18795–18803. 10.1074/jbc.m101382200 11279200

[B100] Meeker R. B. Swanson D. J. Hayward J. N. (1989). Light and electron microscopic localization of glutamate immunoreactivity in the supraoptic nucleus of the rat hypothalamus. Neuroscience 33, 157–167. 10.1016/0306-4522(89)90318-7 2574834

[B101] Minatohara K. Ichikawa S. H. Seki T. Fujiyoshi Y. Doi T. (2013). Ligand binding of PDZ domains has various roles in the synaptic clustering of SAP102 and PSD-95. Neurosci. Lett. 533, 44–49. 10.1016/j.neulet.2012.11.019 23178474

[B102] Mok H. Shin H. Kim S. Lee J. R. Yoon J. Kim E. (2002). Association of the kinesin superfamily motor protein KIF1Balpha with postsynaptic density-95 (PSD-95), synapse-associated protein-97 and synaptic scaffolding molecule PSD-95/discs large/zona occludens-1 proteins. J. Neurosci. 22, 5253–5258. 12097473 10.1523/JNEUROSCI.22-13-05253.2002 PMC6758229

[B103] Mu Y. Otsuka T. Horton A. C. Scott D. B. Ehlers M. D. (2003). Activity-dependent mRNA splicing controls ER export and synaptic delivery of NMDA receptors. Neuron 40, 581–594. 10.1016/s0896-6273(03)00676-7 14642281

[B104] Müller B. M. Kistner U. Kindler S. Chung W. J. Kuhlendahl S. Fenster S. D.. (1996). SAP102, a novel postsynaptic protein that interacts with NMDA receptor complexes in vivo. Neuron 17, 255–265. 10.1016/s0896-6273(00)80157-9 8780649

[B105] Müller B. M. Kistner U. Veh R. W. Cases-Langhoff C. Becker B. Gundelfinger E. D.. (1995). Molecular characterization and spatial distribution of SAP97, a novel presynaptic protein homologous to SAP90 and the Drosophila discs-large tumor suppressor protein. J. Neurosci. 15, 2354–2366. 7891172 10.1523/JNEUROSCI.15-03-02354.1995 PMC6578138

[B106] Nakagawa T. Futai K. Lashuel H. A. Lo I. Okamoto K. Walz T.. (2004). Quaternary structure, protein dynamics and synaptic function of SAP97 controlled by L27 domain interactions. Neuron 44, 453–467. 10.1016/j.neuron.2004.10.012 15504326

[B107] Ng D. Pitcher G. M. Szilard R. K. Sertié A. Kanisek M. Clapcote S. J.. (2009). Neto1 is a novel CUB-domain NMDA receptor-interacting protein required for synaptic plasticity and learning. PLoS Biol. 7:e41. 10.1371/journal.pbio.1000041 19243221 PMC2652390

[B108] Niethammer M. Kim E. Sheng M. (1996). Interaction between the C terminus of NMDA receptor subunits and multiple members of the PSD-95 family of membrane-associated guanylate kinases. J. Neurosci. 16, 2157–2163. 8601796 10.1523/JNEUROSCI.16-07-02157.1996 PMC6578538

[B109] Nilsson A. Eriksson M. Muly E. C. Åkesson E. Samuelsson E.-B. Bogdanovic N.. (2007). Analysis of NR3A receptor subunits in human native NMDA receptors. Brain Res. 1186, 102–112. 10.1016/j.brainres.2007.09.008 17997397

[B110] Okabe S. Miwa A. Okado H. (1999). Alternative splicing of the C-terminal domain regulates cell surface expression of the NMDA receptor NR1 subunit. J. Neurosci. 19, 7781–7792. 10479681 10.1523/JNEUROSCI.19-18-07781.1999 PMC6782467

[B111] Oshima S. Fukaya M. Masabumi N. Shirakawa T. Oguchi H. Watanabe M. (2002). Early onset of NMDA receptor GluRε1 (NR2A) expression and its abundant postsynaptic localization in developing motoneurons of the mouse hypoglossal nucleus. Neurosci. Res. 43, 239–250. 10.1016/s0168-0102(02)00035-4 12103442

[B112] Osterweil E. Wells D. G. Mooseker M. S. (2005). A role for myosin VI in postsynaptic structure and glutamate receptor endocytosis. J. Cell Biol. 168, 329–338. 10.1083/jcb.200410091 15657400 PMC2171578

[B113] Ozaki M. Sasner M. Yano R. Lu H. S. Buonanno A. (1997). Neuregulin-beta induces expression of an NMDA-receptor subunit. Nature 390, 691–694. 9414162 10.1038/37795

[B114] Pabba M. Wong A. Y. Ahlskog N. Hristova E. Biscaro D. Nassrallah W.. (2014). NMDA receptors are upregulated and trafficked to the plasma membrane after Sigma-1 receptor activation in the rat hippocampus. J. Neurosci. 34, 11325–11338. 10.1523/JNEUROSCI.0458-14.2014 25143613 PMC6615506

[B115] Paoletti P. (2011). Molecular basis of NMDA receptor functional diversity. Eur. J. Neurosci. 33, 1351–1365. 10.1111/j.1460-9568.2011.07628.x 21395862

[B116] Paoletti P. Bellone C. Zhou Q. (2013). NMDA receptor subunit diversity: impact on receptor properties, synaptic plasticity and disease. Nat. Rev. Neurosci. 14, 383–400. 10.1038/nrn3504 23686171

[B117] Papadakis M. Hawkins L. M. Stephenson F. A. (2004). Appropriate NR1-NR1 disulfide-linked homodimer formation is requisite for efficient expression of functional, cell surface N-methyl-D-aspartate NR1/NR2 receptors. J. Biol. Chem. 279, 14703–14712. 10.1074/jbc.m313446200 14732708

[B118] Papouin T. Ladépêche L. Ruel J. Sacchi S. Labasque M. Hanini M.. (2012). Synaptic and extrasynaptic NMDA receptors are gated by different endogenous coagonists. Cell 150, 633–646. 10.1016/j.cell.2012.06.029 22863013

[B119] Parsons M. P. Raymond L. A. (2014). Extrasynaptic NMDA receptor involvement in central nervous system disorders. Neuron 82, 279–293. 10.1016/j.neuron.2014.03.030 24742457

[B120] Peng Y. Zhao J. Gu Q. H. Chen R. Q. Xu Z. Yan J. Z.. (2010). Distinct trafficking and expression mechanisms underlie LTP and LTD of NMDA receptor-mediated synaptic responses. Hippocampus 20, 646–658. 10.1002/hipo.20654 19489005

[B121] Penn A. C. Williams S. R. Greger I. H. (2008). Gating motions underlie AMPA receptor secretion from the endoplasmic reticulum. EMBO J. 27, 3056–3068. 10.1038/emboj.2008.222 18923416 PMC2585171

[B122] Pérez-Otaño I. Luján R. Tavalin S. J. Plomann M. Modregger J. Liu X. B.. (2006). Endocytosis and synaptic removal of NR3A-containing NMDA receptors by PACSIN1/syndapin1. Nat. Neurosci. 9, 611–621. 10.1038/nn1680 16617342 PMC1892311

[B123] Perez-Otano I. Schulteis C. T. Contractor A. Lipton S. A. Trimmer J. S. Sucher N. J.. (2001). Assembly with the NR1 subunit is required for surface expression of NR3A-containing NMDA receptors. J. Neurosci. 21, 1228–1237. 11160393 10.1523/JNEUROSCI.21-04-01228.2001 PMC6762235

[B124] Petralia R. S. (2012). Distribution of extrasynaptic NMDA receptors on neurons. ScientificWorldJournal 2012:267120. 10.1100/2012/267120 22654580 PMC3361219

[B125] Petralia R. S. Al-Hallaq R. A. Wenthold R. J. (2009). “Trafficking and targeting of NMDA receptors,” in Biology of the NMDA Receptor, ed Van Dongen A. M. (Boca Raton, FL: Taylor and Francis Group), 149–200. 21204421

[B126] Petralia R. S. Sans N. Wang Y. X. Wenthold R. J. (2005). Ontogeny of postsynaptic density proteins at glutamatergic synapses. Mol. Cell. Neurosci. 29, 436–452. 10.1016/j.mcn.2005.03.013 15894489 PMC1414063

[B129] Petralia R. S. Wang Y. X. Hua F. Yi Z. Zhou A. Ge L.. (2010). Organization of NMDA receptors at extrasynaptic locations. Neuroscience 167, 68–87. 10.1016/j.neuroscience.2010.01.022 20096331 PMC2840201

[B127] Petralia R. S. Wang Y. X. Wenthold R. J. (2002). NMDA receptors and PSD-95 are found in attachment plaques in cerebellar granular layer glomeruli. Eur. J. Neurosci. 15, 583–587. 10.1046/j.1460-9568.2002.01896.x 11876787

[B128] Petralia R. S. Wang Y. X. Wenthold R. J. (2003). Internalization at glutamatergic synapses during development. Eur. J. Neurosci. 18, 3207–3217. 10.1111/j.1460-9568.2003.03074.x 14686895

[B130] Petralia R. S. Wenthold R. J. (2008). “NMDA receptors,” in The Glutamate Receptors, eds Gereau R. W. Swanson G. T. (Totowa, NJ: Humana Press), 45–98.

[B131] Piguel N. H. Fievre S. Blanc J.-M. Carta M. Moreau M. M. Moutin E.. (2014). Scribble1/AP2 complex coordinates NMDA receptor endocytic recycling. Cell Rep. [Epub ahead of print]. S2211-1247(14)00785-2. 10.1016/j.celrep.2014.09.017 25310985

[B132] Priel A. Selak S. Lerma J. Stern-Bach Y. (2006). Block of kainate receptor desensitization uncovers a key trafficking checkpoint. Neuron 52, 1037–1046. 10.1016/j.neuron.2006.12.006 17178406

[B133] Prybylowski K. Chang K. Sans N. Kan L. Vicini S. Wenthold R. J. (2005). The synaptic localization of NR2B-containing NMDA receptors is controlled by interactions with PDZ proteins and AP-2. Neuron 47, 845–857. 10.1016/j.neuron.2005.08.016 16157279 PMC1350965

[B134] Qiu S. Hua Y. L. Yang F. Chen Y. Z. Luo J. H. (2005). Subunit assembly of N-methyl-d-aspartate receptors analyzed by fluorescence resonance energy transfer. J. Biol. Chem. 280, 24923–24930. 10.1074/jbc.m413915200 15888440

[B135] Qiu S. Zhang X. M. Cao J. Y. Yang W. Yan Y. G. Shan L.. (2009). An endoplasmic reticulum retention signal located in the extracellular amino-terminal domain of the NR2A subunit of N-Methyl-D-aspartate receptors. J. Biol. Chem. 284, 20285–20298. 10.1074/jbc.M109.004960 19487695 PMC2740454

[B136] Quinlan E. M. Olstein D. H. Bear M. F. (1999a). Bidirectional, experience-dependent regulation of N-methyl-D-aspartate receptor subunit composition in the rat visual cortex during postnatal development. Proc. Natl. Acad. Sci. U S A 96, 12876–12880. 10.1073/pnas.96.22.12876 10536016 PMC23143

[B137] Quinlan E. M. Philpot B. D. Huganir R. L. Bear M. F. (1999b). Rapid, experience-dependent expression of synaptic NMDA receptors in visual cortex in vivo. Nat. Neurosci. 2, 352–357. 10.1038/7263 10204542

[B138] Rácz B. Blanpied T. A. Ehlers M. D. Weinberg R. J. (2004). Lateral organization of endocytic machinery in dendritic spines. Nat. Neurosci. 7, 917–918. 10.1038/nn1303 15322548

[B139] Rebola N. Srikumar B. N. Mulle C. (2010). Activity-dependent synaptic plasticity of NMDA receptors. J. Physiol. 588, 93–99. 10.1113/jphysiol.2009.179382 19822542 PMC2821550

[B140] Riefler G. M. Balasingam G. Lucas K. G. Wang S. Hsu S. C. Firestein B. L. (2003). Exocyst complex subunit sec8 binds to postsynaptic density protein-95 (PSD-95): a novel interaction regulated by cypin (cytosolic PSD-95 interactor). Biochem. J. 373, 49–55. 10.1042/bj20021838 12675619 PMC1223477

[B141] Roberts A. C. Díez-Garcia J. Rodriguiz R. M. López I. P. Luján R. Martínez-Turrillas R.. (2009). Downregulation of NR3A-containing NMDARs is required for synapse maturation and memory consolidation. Neuron 63, 342–356. 10.1016/j.neuron.2009.06.016 19679074 PMC3448958

[B142] Roche K. W. Standley S. McCallum J. Dune Ly C. Ehlers M. D. Wenthold R. J. (2001). Molecular determinants of NMDA receptor internalization. Nat. Neurosci. 4, 794–802. 10.1038/90498 11477425

[B143] Rodenas-Ruano A. Chávez A. E. Cossio M. J. Castillo P. E. Zukin R. S. (2012). REST-dependent epigenetic remodeling promotes the developmental switch in synaptic NMDA receptors. Nat. Neurosci. 15, 1382–1390. 10.1038/nn.3214 22960932 PMC3501125

[B144] Salussolia C. L. Corrales A. Talukder I. Kazi R. Akgul G. Bowen M.. (2011). Interaction of the M4 segment with other transmembrane segments is required for surface expression of mammalian AMPA receptors. J. Biol. Chem. 286, 40205–40218. 10.1074/jbc.m111.268839 21930708 PMC3220572

[B145] Salussolia C. L. Gan Q. Kazi R. Singh P. Allopenna J. Furukawa H.. (2013). A eukaryotic specific transmembrane segment is required for tetramerization in AMPA receptors. J. Neurosci. 33, 9840–9845. 10.1523/JNEUROSCI.2626-12.2013 23739980 PMC3714855

[B146] Sans N. Petralia R. S. Wang Y. X. Blahos J. 2nd Hell J. W. Wenthold R. J. (2000). A developmental change in NMDA receptor-associated proteins at hippocampal synapses. J. Neurosci. 20, 1260–1271. 10648730 10.1523/JNEUROSCI.20-03-01260.2000 PMC6774158

[B147] Sans N. Prybylowski K. Petralia R. S. Chang K. Wang Y. X. Racca C.. (2003). NMDA receptor trafficking through an interaction between PDZ proteins and the exocyst complex. Nat. Cell Biol. 5, 520–530. 10.1038/ncb990 12738960

[B148] Sans N. Racca C. Petralia R. S. Wang Y. X. McCallum J. Wenthold R. J. (2001). Synapse-associated protein 97 selectively associates with a subset of AMPA receptors early in their biosynthetic pathway. J. Neurosci. 21, 7506–7516. 11567040 10.1523/JNEUROSCI.21-19-07506.2001 PMC6762903

[B149] Sans N. Wang P. Y. Du Q. Petralia R. S. Wang Y. X. Nakka S.. (2005). mPins modulates PSD-95 and SAP102 trafficking and influences NMDA receptor surface expression. Nat. Cell Biol. 7, 1179–1190. 10.1038/ncb1325 16299499

[B150] Sanz-Clemente A. Gray J. A. Ogilvie K. A. Nicoll R. A. Roche K. W. (2013a). Activated CaMKII couples GluN2B and casein kinase 2 to control synaptic NMDA receptors. Cell Rep. 3, 607–614. 10.1016/j.celrep.2013.02.011 23478024 PMC3615108

[B151] Sanz-Clemente A. Matta J. A. Isaac J. T. Roche K. W. (2010). Casein kinase 2 regulates the NR2 subunit composition of synaptic NMDA receptors. Neuron 67, 984–996. 10.1016/j.neuron.2010.08.011 20869595 PMC2947143

[B152] Sanz-Clemente A. Nicoll R. A. Roche K. W. (2013b). Diversity in NMDA receptor composition: many regulators, many consequences. Neuroscientist 19, 62–75. 10.1177/1073858411435129 22343826 PMC3567917

[B153] Schlüter O. M. Xu W. Malenka R. C. (2006). Alternative N-terminal domains of PSD-95 and SAP97 govern activity-dependent regulation of synaptic AMPA receptor function. Neuron 51, 99–111. 10.1016/j.neuron.2006.05.016 16815335

[B154] Schorge S. Colquhoun D. (2003). Studies of NMDA receptor function and stoichiometry with truncated and tandem subunits. J. Neurosci. 23, 1151–1158. 12598603 10.1523/JNEUROSCI.23-04-01151.2003 PMC6742241

[B155] Schüler T. Mesic I. Madry C. Bartholomäus I. Laube B. (2008). Formation of NR1/NR2 and NR1/NR3 heterodimers constitutes the initial step in N-methyl-D-aspartate receptor assembly. J. Biol. Chem. 283, 37–46. 10.1074/jbc.m703539200 17959602

[B156] Scott D. B. Blanpied T. A. Swanson G. T. Zhang C. Ehlers M. D. (2001). An NMDA receptor ER retention signal regulated by phosphorylation and alternative splicing. J. Neurosci. 21, 3063–3072. 11312291 10.1523/JNEUROSCI.21-09-03063.2001 PMC6762585

[B157] Seaman M. N. Gautreau A. Billadeau D. D. (2013). Retromer-mediated endosomal protein sorting: all WASHed up! Trends Cell Biol. 23, 522–528. 10.1016/j.tcb.2013.04.010 23721880 PMC3924425

[B158] Setou M. Nakagawa T. Seog D. H. Hirokawa N. (2000). Kinesin superfamily motor protein KIF17 and mLin-10 in NMDA receptor-containing vesicle transport. Science 288, 1796–1802. 10.1126/science.288.5472.1796 10846156

[B159] She K. Ferreira J. S. Carvalho A. L. Craig A. M. (2012). Glutamate binding to the GluN2B subunit controls surface trafficking of N-methyl-D-aspartate (NMDA) receptors. J. Biol. Chem. 287, 27432–27445. 10.1074/jbc.M112.345108 22740692 PMC3431666

[B160] Sheng M. (1996). PDZs and receptor/channel clustering: rounding up the latest suspects. Neuron 17, 575–578. 10.1016/s0896-6273(00)80190-7 8893015

[B161] Sheng M. Cummings J. Roldan L. A. Jan Y. N. Jan L. Y. (1994). Changing subunit composition of heteromeric NMDA receptors during development of rat cortex. Nature 368, 144–147. 10.1038/368144a0 8139656

[B162] Sheng M. Kim E. (1996). Ion channel associated proteins. Curr. Opin. Neurobiol. 6, 602–608. 10.1016/s0959-4388(96)80091-2 8937823

[B163] Shinohara Y. Hirase H. Watanabe M. Itakura M. Takahashi M. Shigemoto R. (2008). Left-right asymmetry of the hippocampal synapses with differential subunit allocation of glutamate receptors. Proc. Natl. Acad. Sci. U S A 105, 19498–19503. 10.1073/pnas.0807461105 19052236 PMC2593619

[B164] Siegler Retchless B. Gao W. Johnson J. W. (2012). A single GluN2 subunit residue controls NMDA receptor channel properties via intersubunit interaction. Nat. Neurosci. 15, 406–413. 10.1038/nn.3025 22246434 PMC3288527

[B165] Songyang Z. Fanning A. S. Fu C. Xu J. Marfatia S. M. Chishti A. H.. (1997). Recognition of unique carboxyl-terminal motifs by distinct PDZ domains. Science 275, 73–77. 10.1126/science.275.5296.73 8974395

[B166] Standley S. Petralia R. S. Gravell M. Hamilton R. Wang Y. X. Schubert M.. (2012). Trafficking of the NMDAR2B receptor subunit distal cytoplasmic tail from endoplasmic reticulum to the synapse. PLoS One 7:e39585. 10.1371/journal.pone.0039585 22761831 PMC3384676

[B167] Standley S. Roche K. W. McCallum J. Sans N. Wenthold R. J. (2000). PDZ domain suppression of an ER retention signal in NMDA receptor NR1 splice variants. Neuron 28, 887–898. 10.1016/s0896-6273(00)00161-6 11163274

[B168] Suh Y. H. Terashima A. Petralia R. S. Wenthold R. J. Isaac J. T. Roche K. W.. (2010). A neuronal role for SNAP-23 in postsynaptic glutamate receptor trafficking. Nat. Neurosci. 13, 338–343. 10.1038/nn.2488 20118925 PMC2861127

[B169] Swanwick C. C. Shapiro M. E. Yi Z. Chang K. Wenthold R. J. (2009). NMDA receptors interact with flotillin-1 and -2, lipid raft-associated proteins. FEBS Lett. 583, 1226–1230. 10.1016/j.febslet.2009.03.017 19298817

[B195] Tall G. G. Gilman A. G. (2005). Resistance to inhibitors of cholinesterase 8A catalyzes release of Galphai-GTP and nuclear mitotic apparatus protein (NuMA) from NuMA/LGN/Galphai-GDP complexes. Proc. Natl. Acad. Sci. U S A 102, 16584–16589. 10.1073/pnas.0508306102 16275912 PMC1283842

[B170] Thomas C. G. Miller A. J. Westbrook G. L. (2006). Synaptic and extrasynaptic NMDA receptor NR2 subunits in cultured hippocampal neurons. J. Neurophysiol. 95, 1727–1734. 10.1152/jn.00771.2005 16319212

[B171] Tovar K. R. McGinley M. J. Westbrook G. L. (2013). Triheteromeric NMDA receptors at hippocampal synapses. J. Neurosci. 33, 9150–9160. 10.1523/JNEUROSCI.0829-13.2013 23699525 PMC3755730

[B172] Tovar K. R. Westbrook G. L. (1999). The incorporation of NMDA receptors with a distinct subunit composition at nascent hippocampal synapses in vitro. J. Neurosci. 19, 4180–4188. 10234045 10.1523/JNEUROSCI.19-10-04180.1999 PMC6782704

[B173] Tovar K. R. Westbrook G. L. (2002). Mobile NMDA receptors at hippocampal synapses. Neuron 34, 255–264. 10.1016/s0896-6273(02)00658-x 11970867

[B174] Traynelis S. F. Wollmuth L. P. McBain C. J. Menniti F. S. Vance K. M. Ogden K. K.. (2010). Glutamate receptor ion channels: structure, regulation and function. Pharmacol. Rev. 62, 405–496. 10.1124/pr.109.002451 20716669 PMC2964903

[B175] Vissel B. Krupp J. J. Heinemann S. F. Westbrook G. L. (2001). A use-dependent tyrosine dephosphorylation of NMDA receptors is independent of ion flux. Nat. Neurosci. 4, 587–596. 10.1038/88404 11369939

[B176] Wang P. Y. Petralia R. S. Wang Y. X. Wenthold R. J. Brenowitz S. D. (2011). Functional NMDA receptors at axonal growth cones of young hippocampal neurons. J. Neurosci. 31, 9289–9297. 10.1523/JNEUROSCI.5639-10.2011 21697378 PMC3124703

[B177] Wang X. Zhao Y. Zhang X. Badie H. Zhou Y. Mu Y.. (2013). Loss of sorting nexin 27 contributes to excitatory synaptic dysfunction by modulating glutamate receptor recycling in down’s syndrome. Nat. Med. 19, 473–480. 10.1038/nm.3117 23524343 PMC3911880

[B178] Washbourne P. Bennett J. E. McAllister A. K. (2002). Rapid recruitment of NMDA receptor transport packets to nascent synapses. Nat. Neurosci. 5, 751–759. 10.1038/nn883 12089529

[B179] Washbourne P. Liu X. B. Jones E. G. McAllister A. K. (2004). Cycling of NMDA receptors during trafficking in neurons before synapse formation. J. Neurosci. 24, 8253–8264. 10.1523/jneurosci.2555-04.2004 15385609 PMC6729693

[B180] Wenthold R. J. Prybylowski K. Standley S. Sans N. Petralia R. S. (2003). Trafficking of NMDA receptors. Annu. Rev. Pharmacol. Toxicol. 43, 335–358. 10.1146/annurev.pharmtox.43.100901.135803 12540744

[B181] Wong H. K. Liu X. B. Matos M. F. Chan S. F. Pérez-Otaño I. Boysen M.. (2002). Temporal and regional expression of NMDA receptor subunit NR3A in the mammalian brain. J. Comp. Neurol. 450, 303–317. 10.1002/cne.10314 12209845

[B182] Wu H. Nash J. E. Zamorano P. Garner C. C. (2002). Interaction of SAP97 with minus-end-directed actin motor myosin VI. Implications for AMPA receptor trafficking. J. Biol. Chem. 277, 30928–30934. 10.1074/jbc.m203735200 12050163

[B183] Wyeth M. S. Pelkey K. A. Petralia R. S. Salter M. W. McInnes R. R. McBain C. J. (2014). Neto auxiliary protein interactions regulate kainate and NMDA receptor subunit localization at mossy fiber-CA3 pyramidal cell synapses. J. Neurosci. 34, 622–628. 10.1523/JNEUROSCI.3098-13.2014 24403160 PMC3870939

[B184] Xu Z. Chen R. Q. Gu Q. H. Yan J. Z. Wang S. H. Liu S. Y.. (2009). Metaplastic regulation of long-term potentiation/long-term depression threshold by activity-dependent changes of NR2A/NR2B ratio. J. Neurosci. 29, 8764–8773. 10.1523/JNEUROSCI.1014-09.2009 19587283 PMC6664898

[B185] Yang W. Zheng C. Song Q. Yang X. Qiu S. Liu C.. (2007). A three amino acid tail following the TM4 region of the N-methyl-D-aspartate receptor (NR) 2 subunits is sufficient to overcome endoplasmic reticulum retention of NR1–1a subunit. J. Biol. Chem. 282, 9269–9278. 10.1074/jbc.m700050200 17255096

[B186] Yashiro K. Corlew R. Philpot B. D. (2005). Visual deprivation modifies both presynaptic glutamate release and the composition of perisynaptic/extrasynaptic NMDA receptors in adult visual cortex. J. Neurosci. 25, 11684–11692. 10.1523/jneurosci.4362-05.2005 16354927 PMC6726025

[B187] Yi Z. Petralia R. S. Fu Z. Swanwick C. C. Wang Y. X. Prybylowski K.. (2007). The role of the PDZ protein GIPC in regulating NMDA receptor trafficking. J. Neurosci. 27, 11663–11675. 10.1523/jneurosci.3252-07.2007 17959809 PMC6673220

[B188] Yin X. Takei Y. Kido M. A. Hirokawa N. (2011). Molecular motor KIF17 is fundamental for memory and learning via differential support of synaptic NR2A/2B levels. Neuron 70, 310–325. 10.1016/j.neuron.2011.02.049 21521616

[B189] Yoshii A. Sheng M. H. Constantine-Paton M. (2003). Eye opening induces a rapid dendritic localization of PSD-95 in central visual neurons. Proc. Natl. Acad. Sci. U S A 100, 1334–1339. 10.1073/pnas.0335785100 12552131 PMC298773

[B190] Yuan T. Mameli M. O’Connor E. C. Dey P. N. Verpelli C. Sala C.. (2013). Expression of cocaine-evoked synaptic plasticity by GluN3A-containing NMDA receptors. Neuron 80, 1025–1038. 10.1016/j.neuron.2013.07.050 24183704

[B191] Zhang J. Diamond J. S. (2009). Subunit- and pathway-specific localization of NMDA receptors and scaffolding proteins at ganglion cell synapses in rat retina. J. Neurosci. 29, 4274–4286. 10.1523/JNEUROSCI.5602-08.2009 19339621 PMC4283557

[B192] Zhao J. Peng Y. Xu Z. Chen R. Q. Gu Q. H. Chen Z.. (2008). Synaptic metaplasticity through NMDA receptor lateral diffusion. J. Neurosci. 28, 3060–3070. 10.1523/JNEUROSCI.5450-07.2008 18354009 PMC6670713

[B193] Zheng C. Y. Petralia R. S. Wang Y. X. Kachar B. Wenthold R. J. (2010). SAP102 is a highly mobile MAGUK in spines. J. Neurosci. 30, 4757–4766. 10.1523/JNEUROSCI.6108-09.2010 20357126 PMC2874826

[B194] Zheng C. Y. Seabold G. K. Horak M. Petralia R. S. (2011). MAGUKs, synaptic development and synaptic plasticity. Neuroscientist 17, 493–512. 10.1177/1073858410386384 21498811 PMC3191319

